# Atomically Precise Carbyne-Nanoribbon Devices: Transmission Engineering through Ring Topology and Contact-Site Selection

**DOI:** 10.1021/acsomega.6c05188

**Published:** 2026-07-24

**Authors:** Ana Beatriz Rosa da Silva, Iurbson Sales Costa, Alessandre Sampaio-Silva, J. Del Nero

**Affiliations:** † Departamento de Física, 306972 UEPA, Belém 66050-540, Brazil; ‡ Faculdade de Física, 37871 UFPA, Belém 66075-110, Brazil

## Abstract

We present a first-principles density functional theory combined with the nonequilibrium Green’s function formalism (DFT/NEGF) investigation of charge transport in eight molecular junction configurations formed by graphene and phagraphene nanoribbons with zigzag and armchair edge topologies connected to semi-infinite sp-hybridized carbyne chain electrodes at two geometrically distinct attachment sites. In the first contact geometry, the carbyne electrode bonds to an sp^2^ carbon atom sharing its ring connectivity with one adjacent hexagonal ring (referred to as the edge-proximal site), whereas in the second contact geometry, the electrode bonds to an atom whose ring environment includes a pentagonal ring of the 5–6–7 phagraphene network or an equivalent laterally shifted hexagonal site (referred to as the extended-conjugation site). Systematic variation of ring topology, edge orientation, and contact geometry isolates their individual contributions to the quantum transport response. The electrode attachment site is the primary qualitative determinant: the edge-proximal attachment produces weakly conducting, rectifying junctions governed by sharp transmission resonances, while the extended-conjugation attachment opens high-transmission pathways. Ring topology provides the critical quantitative modulation: the zigzag phagraphene junction at the extended-conjugation site carries −27.5 to +28.3 μA at ±1.0 V with a rectification ratio of 1.03 and zero-bias conductance of 0.37 G_0_, a 3.3-fold enhancement over its graphene counterpart. The armchair phagraphene device at the edge-proximal site exhibits voltage-controlled rectification reaching a ratio of 4.2 at ±0.5 V, arising from a near-resonant Fermi-level state with a density of states up to 356 eV^–1^ created by the 5–6–7 ring topology. These results establish quantitative design rules linking ring topology, edge orientation, and contact geometry to conductance and rectification in sp–sp^2^ carbon nanoelectronic junctions.

## Graphical abstract

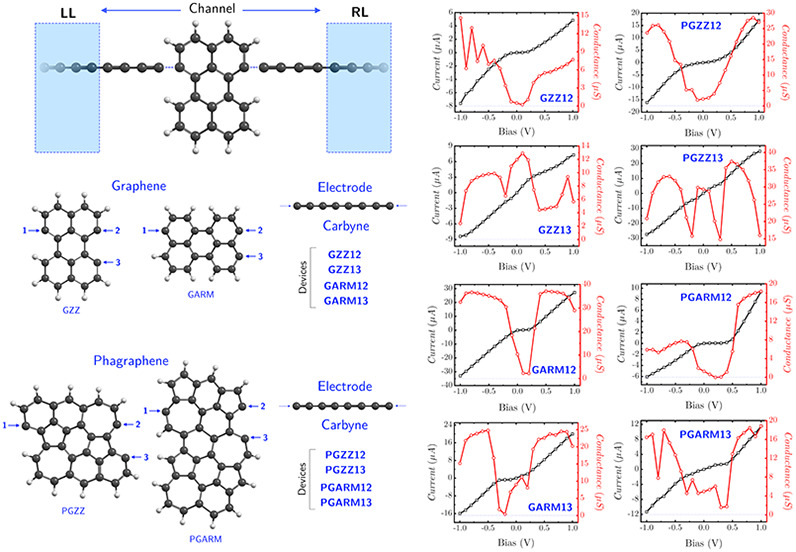

## 1 Introduction

Carbon’s structural versatility spans hybridization states from three-dimensional sp^3^ diamond to two-dimensional sp^2^ graphene and one-dimensional sp carbyne, making it uniquely suited as a single-element platform for atomically precise nanoelectronics.^1–3^ Among two-dimensional allotropes, graphene nanoribbons (GNRs) with armchair and zigzag edge topologies display strikingly different electronic characters, including width-dependent semiconducting gaps vs localized magnetic edge states, and bottom-up on-surface synthesis has made atomically precise ribbons experimentally accessible with confirmed transistor action and topological in-gap states.^4–10^ Beyond the purely hexagonal network of graphene, phagraphene, a 2D sp^2^ allotrope built from alternating pentagonal, hexagonal, and heptagonal rings, hosts distorted Dirac cones and supports width-dependent topological insulator-to-metal transitions in nanoribbon form.^11,12^ Related nonhexagonal sp^2^ networks, including biphenylene (4–6–8 rings), have already been realized experimentally by on-surface synthesis,^13^ establishing that 2D carbon networks incorporating nonhexagonal rings are accessible materials with distinct transport properties. Carbyne, the sp-hybridized one-dimensional allotrope, exhibits extraordinary mechanical stiffness and strongly geometry-dependent conductance; its synthesis and stabilization inside carbon nanotubes and by on-surface demetallization routes have advanced markedly.^14–19^

The integration of carbyne chains as atomically defined electrodes contacting 2D nanoribbon scattering regions creates a hybrid sp–sp^2^ junction family whose transport properties depend simultaneously on ring topology, edge orientation, and electrode contact geometry. Prior density functional theory combined with the nonequilibrium Green’s function formalism (DFT/NEGF) studies have addressed these contributions individually: phagraphene nanoribbon width and doping,^12,20^ graphene-phagraphene heterojunctions,^21^ and sp-carbon chain contacts to SWCNT and metal electrodes.^22,23^ However, a systematic study simultaneously varying ring topology (graphene vs phagraphene), edge orientation (zigzag vs armchair), and contact site within a controlled device family has not been reported, leaving the interplay of these degrees of freedom and the emergent design rules unquantified.

Here, we report a first-principles DFT/NEGF study of eight device configurations. By varying the nanoribbon ring composition and electrode attachment while keeping the architecture fixed, we isolate the effects of topology, edge orientation, and contact geometry on transport. A comparison between graphene and phagraphene families provides design rules for molecular-wire, rectifier, and sensing applications in atomically precise sp–sp^2^ carbon nanoelectronics.

The use of sp-carbon chains as electrodes or active components in molecular junctions has accelerated markedly in recent years, driven by the unique electronic properties of carbyne. The most directly relevant recent advance is the experimental demonstration of molecular junctions using polyynes up to ca. 5 nm in length by Gao et al., who used STM break junctions to map the tunneling conductance of pyridyl-end-capped polyynes Py­[n] (n = 4, 6, 8, 10, 12, 16) and probe the limit of the tunneling regime.^24^ The low attenuation factor they report for long polyynes is consistent with our theoretical prediction of near-unity transmission T ≈ 1.0 in PGZZ13 at the 1–3 contact site, where the sp-carbyne electrode accesses the most efficient conjugation pathway through the phagraphene scattering region.

Performed DFT-NEGF calculations on bipyridine isomers bridged by polyyne-type carbyne electrodes and found that the presence of nitrogen atoms at the molecule–electrode interface improves electronic coupling, and that carbyne-contacted devices outperform analogous junctions with Au, Ag, Cu, or graphene nanoribbon electrodes.^25^ This finding directly supports our choice of semi-infinite sp-carbyne chain electrodes as atomically defined, high-performance leads, whose coupling to the nanoribbon scattering region is dominated by the local bonding geometry at the contact atom.

The broader context of carbyne-based molecular electronics is established by Corrêa et al. on polyyne bridges to SWCNT electrodes^22^ and by Mota et al., who studied carbyne–DNA–carbyne devices and demonstrated the sensitivity of sp-carbon electrode coupling to molecular structure.^26^ These results collectively establish that sp-carbon chain electrodes provide a uniquely clean, contact-geometry-sensitive probe of molecular junction physics. Our eight-device systematic comparison extends this platform to the sp–sp^2^ hybrid architecture and provides the first quantitative design rules for engineering rectification and conductance in carbyne-nanoribbon junctions.

## 2 Methodology

Electronic structure calculations were performed with SIESTA^27^ using the GGA-PBE exchange-correlation functional,^28^ norm-conserving Troullier-Martins pseudopotentials,^29^ and a double-ζ polarized (DZP) basis set with a 300 Ry real-space cutoff. An electronic temperature of 300 K and a maximum residual force of 0.01 eV/Å were applied throughout. Eight two-probe device configurations (Figure 1) were constructed by connecting zigzag and armchair graphene and phagraphene nanoribbons (labeled GZZ12, GZZ13, GARM12, GARM13, PGZZ12, PGZZ13, PGARM12, and PGARM13) to semi-infinite sp-carbyne chain electrodes at positions 1–2 or 1–3 on each ribbon. In each label, G/PG denotes graphene/phagraphene, ZZ/ARM denotes zigzag/armchair edge topology, and the final two digits identify the left and right electrode attachment positions. In each label, the prefix G denotes that the scattering region is a graphene nanoribbon built from the purely hexagonal sp^2^ carbon lattice, while the prefix PG designates a phagraphene nanoribbon, whose backbone is composed of alternating pentagonal, hexagonal, and heptagonal carbon rings in the 5–6–7 connectivity pattern first predicted by Wang et al. The second field of the label describes the edge topology: ZZ (zigzag) refers to nanoribbons terminated by a sequence of carbon atoms all belonging to the same sublattice, which supports quasi-flat bands and localized electronic edge states near the Fermi level, whereas ARM (armchair) refers to nanoribbons whose edge alternates between the two sublattices, producing quantized transverse subbands and a width-dependent semiconducting gap. The final two digits encode the left and right electrode attachment positions, where position 1 is fixed as the left carbyne contact throughout all configurations, and positions 2 and 3 designate alternative right electrode attachment sites that are geometrically and electronically inequivalent.

**Figure 1:**
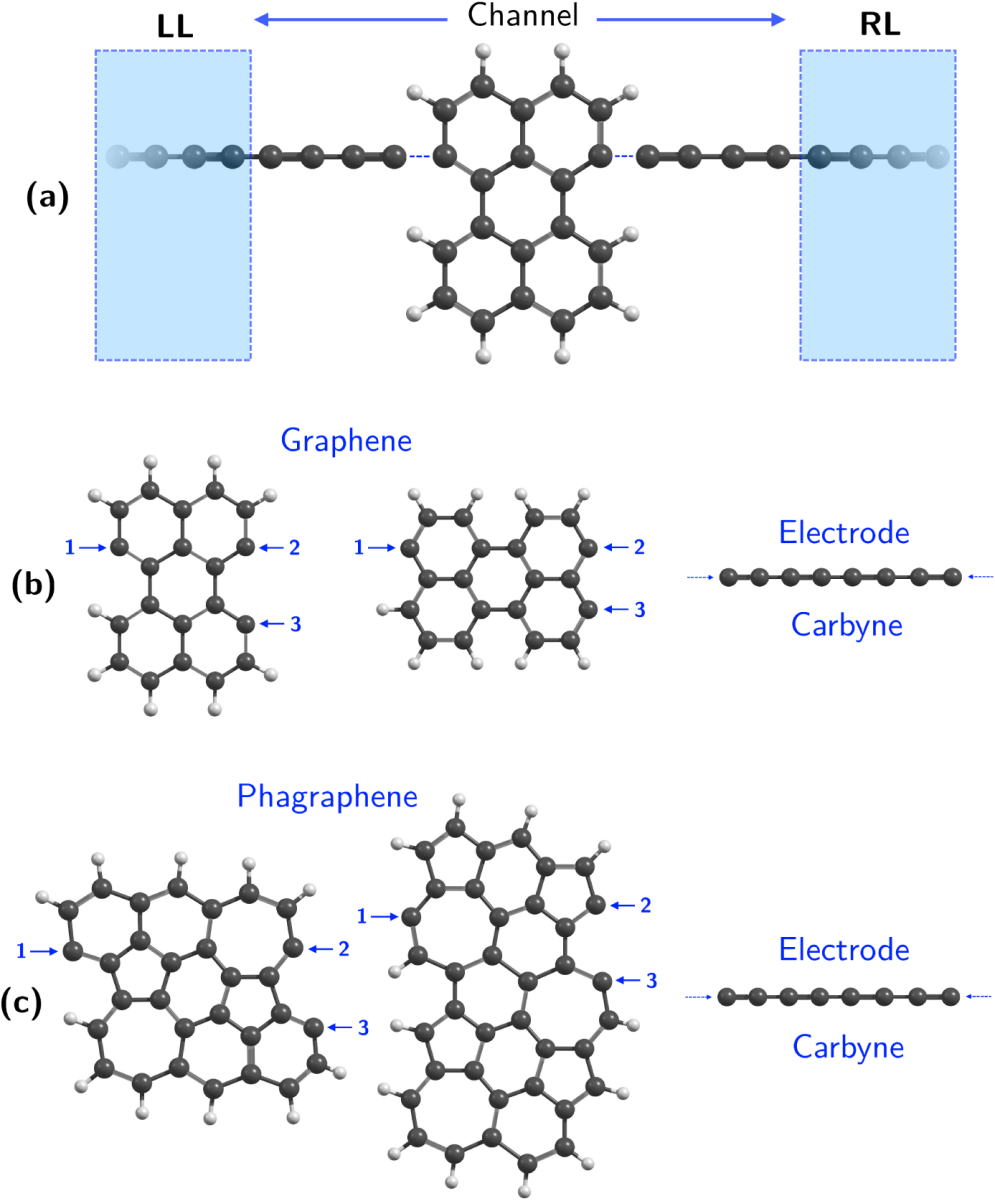
(a) Schematic representation of the two-probe device architecture: the left lead (LL) and right lead (RL) are semi-infinite sp-hybridized carbyne chains symmetrically connected to the nanoribbon scattering region (Channel). (b) Graphene nanoribbon scattering geometries (zigzag, left; armchair, right) with labeled carbyne-electrode attachment positions 1, 2, and 3, and the periodic carbyne electrode unit. (c) Phagraphene nanoribbon scattering geometries (zigzag, left; armchair, right) with the same position labeling convention and the corresponding carbyne electrode unit. Dark gray spheres represent carbon atoms; light gray spheres represent hydrogen edge-passivation atoms throughout.

The distinction between attachment positions 2 and 3 is a critical structural parameter governing the transport response. In the zigzag graphene geometry, position 2 connects the right carbyne electrode to a carbon atom located at the ribbon edge that shares its local ring environment exclusively with neighboring hexagonal rings and whose on-site orbital belongs to the same sublattice as the left electrode anchor. This configuration places the current path along a direction parallel to the edge-localized flat-band states, resulting in a short conjugation path that traverses primarily the region of high edge-state density. Position 3, by contrast, connects the electrode to a carbon atom further into the ribbon interior, displacing the right contact laterally by one bond position relative to position 2 and routing the current through a longer but more delocalized π-conjugation network that spans both sublattices and involves a substantially larger number of contributing π orbitals. The structural consequence of this shift is that the overlap between the transmission eigenfunction of the right electrode and the extended π states of the ribbon is significantly larger at position 3, explaining the 24-fold increase in zero-bias conductance observed in GZZ13 relative to GZZ12.

In the armchair graphene ribbon, position 2 places the right contact at an sp^2^ atom whose local bonding geometry involves two ribbon-transverse C–C bonds directed toward the ribbon interior and one bond along the armchair edge. This creates an asymmetric coupling to the delocalized armchair subbands. Position 3 displaces the contact by one bond along the armchair edge, placing it on an atom whose three bonds are directed with a different angular relationship to the transport axis, altering the symmetry matching between the carbyne π orbital and the armchair subband wave functions. The result is a qualitative reorganization of the transmission function from the high and broadly distributed T­(E,V) surface of GARM12 to the narrow resonance-valley landscape of GARM13, which is the spectroscopic signature of a change in the dominant scattering pathway.

In the phagraphene nanoribbons, the 5–6–7 ring topology introduces a third inequivalent bonding environment at each potential contact atom that has no counterpart in the hexagonal graphene lattice. The pentagon–hexagon–heptagon ring junctions create carbon atoms with three distinct local ring coordination environments: atoms shared between a pentagonal and a hexagonal ring, atoms at the junction of a hexagonal and a heptagonal ring, and atoms belonging exclusively to hexagonal rings. The nonhexagonal ring membership modifies the π-orbital phase relationship at each atom relative to the nearest electrode connection, so that the same macroscopic positional label (2 or 3) at a phagraphene edge atom corresponds to a different microscopic π-coupling path than at the equivalent graphene position. This explains why the introduction of the 5–6–7 connectivity consistently enhances the current magnitude and alters the rectification character even when the contact position label is held fixed.

Quantum transport was calculated self-consistently with TranSIESTA^30^ using the Landauer–Büttiker formalism; k-meshes of 1 × 1 × 100 (transport direction) and 1 × 15 × 1 (transverse) were verified to yield converged transmission functions. I–V curves were obtained at bias voltages from 0 to ±1.0 V in 0.1 V steps. Full equations, convergence benchmarks, and the device nomenclature are given in Section S2 of the Supporting Information.

The semi-infinite carbyne chains employed as left and right electrodes in all eight device configurations are justified by two complementary experimental precedents. First, and most directly relevant, Zhang et al. recently demonstrated the low-temperature synthesis of weakly confined carbyne inside large-diameter single-walled carbon nanotubes by annealing ammonium deoxycholate, obtaining carbyne with a Raman C-mode at 1860–1870 cm^–1^ indicative of a nearly free-standing polyyne character.^19^ In this geometry, the carbyne chain runs continuously through the nanotube interior, and the weak van der Waals interaction between the chain and the nanotube wall introduces minimal hybridization. This configuration closely approximates the semi-infinite electrode model used here, in which the chain extends without defects into the electrode reservoir region, and its electronic structure is that of the optimized free-standing polyyne.

Second, the computational use of semi-infinite sp-carbon electrodes has been validated in prior DFT/NEGF studies from our group. Corrêa et al. demonstrated that polyyne chains connected to semi-infinite SWCNT electrodes yield converged transmission functions and physically meaningful I–V curves that are consistent with experimental conductance measurements on sp-carbon chain junctions.^22^ Ferreira et al. showed that semi-infinite atomic-diameter carbyne chains coupled to nickel electrodes produce well-defined self-energies through the principal-layer decimation technique, and that the resulting transmission and conductance agree quantitatively with tight-binding estimates.^23^ Mota et al. extended this approach to hybrid carbyne–DNA–carbyne devices, confirming that the semi-infinite chain electrode framework is numerically robust and physically transparent for sp-carbon molecular junctions.^26^

Regarding Fermi-level alignment, the carbyne electrodes have their Fermi level set by the self-consistent DFT/NEGF calculation, which places the electrode chemical potential at the GGA-PBE Fermi energy of the optimized periodic carbyne chain. The partial Peierls gap of the polyyne electrode means that the transmission function of the isolated electrode is not constant near the Fermi level, and this feature is fully incorporated in the self-energy matrices through the surface Green’s function calculation via the principal-layer decimation technique.^29,30^ The coupling between the carbyne electrode and the nanoribbon sp^2^ atom at each contact site is determined self-consistently from the DFT Hamiltonian of the optimized junction geometry, and no empirical coupling parameters are introduced. This approach is consistent with the methodology validated for sp-carbon chain junctions in the literature.^22,23,26^

## 3 Results and Discussion

### 3.1 Electronic Structure Background

The transport landscape of these devices is shaped by three distinct electronic subsystems. Graphene nanoribbons contribute either quasi-flat near-Fermi bands (zigzag, producing sharp DOS peaks characteristic of localized edge states^31,32^ ) or quantized subband gaps (armchair, governed by quantum confinement^4,5^ ). Phagraphene nanoribbons carry the same qualitative distinction by edge orientation but with a richer width-dependent phase diagram arising from the 5–6–7 ring connectivity, which breaks the 6-fold rotational symmetry and distorts the Dirac cones.^11,12^ The sp-carbyne electrodes present a partially Peierls-gapped transmission function that selectively couples to nanoribbon states depending on the local bonding geometry of the contact atom, establishing the structural basis for the transport anisotropy documented below.^14,16,22,23,26^ Band structure details for all three subsystems are provided in Section S3 of the Supporting Information.

### 3.2 Transport Characteristics

The I–V characteristics, differential conductance (dI/dV), bias-dependent transmission T­(E,V), and density of states DOS­(E,V), for all eight configurations are presented in Figures 2 and 3 for the graphene zigzag and armchair families, respectively, and in Figures 4 and 5 for the phagraphene zigzag and armchair families. A summary of key transport metrics for all eight configurations is provided in Table 1.

**Table 1:** Summary of Key Zero-Bias Transport Metrics for All Eight Carbyne-Nanoribbon Device Configurations^a^

Config.	Zero-bias conductance	I at +1 V (μA)	I at –1 V (μA)	Max rect. ratio	DOS peak (eV^–1^)
GZZ12	∼0.01 G_0_ (0.5 μS)	+4.85	–7.62	1.57 at ±1.0 V	60
GZZ13	0.15 G_0_ (12 μS)	+7.29	–8.40	1.15 at ±1.0 V	50
GARM12	>0.2 G_0_	+27.2	–32.9	1.21 at ±1.0 V	1.6
GARM13	∼0.1 G_0_	+20.0	–16.0	1.25 at ±1.0 V	400
PGZZ12	0.029 G_0_ (2.3 μS)	+17.0	–16.1	1.06 at ±1.0 V	70
PGZZ13	0.37 G_0_ (29 μS)	+28.3	–27.5	1.03 at ±1.0 V	∼50
PGARM12	∼0.01 G_0_	+9.38	–6.07	4.2 at ±0.5 V	356
PGARM13	∼0.08 G_0_	+11.5	–11.3	1.02 at ±1.0 V	90

a G_0_ = 2e^2^/h = 77.5 μs is the quantum of conductance. Rectification ratio is defined as |I­(−V)|/|I­(+V)| or its inverse, whichever exceeds unity.

**Figure 2:**
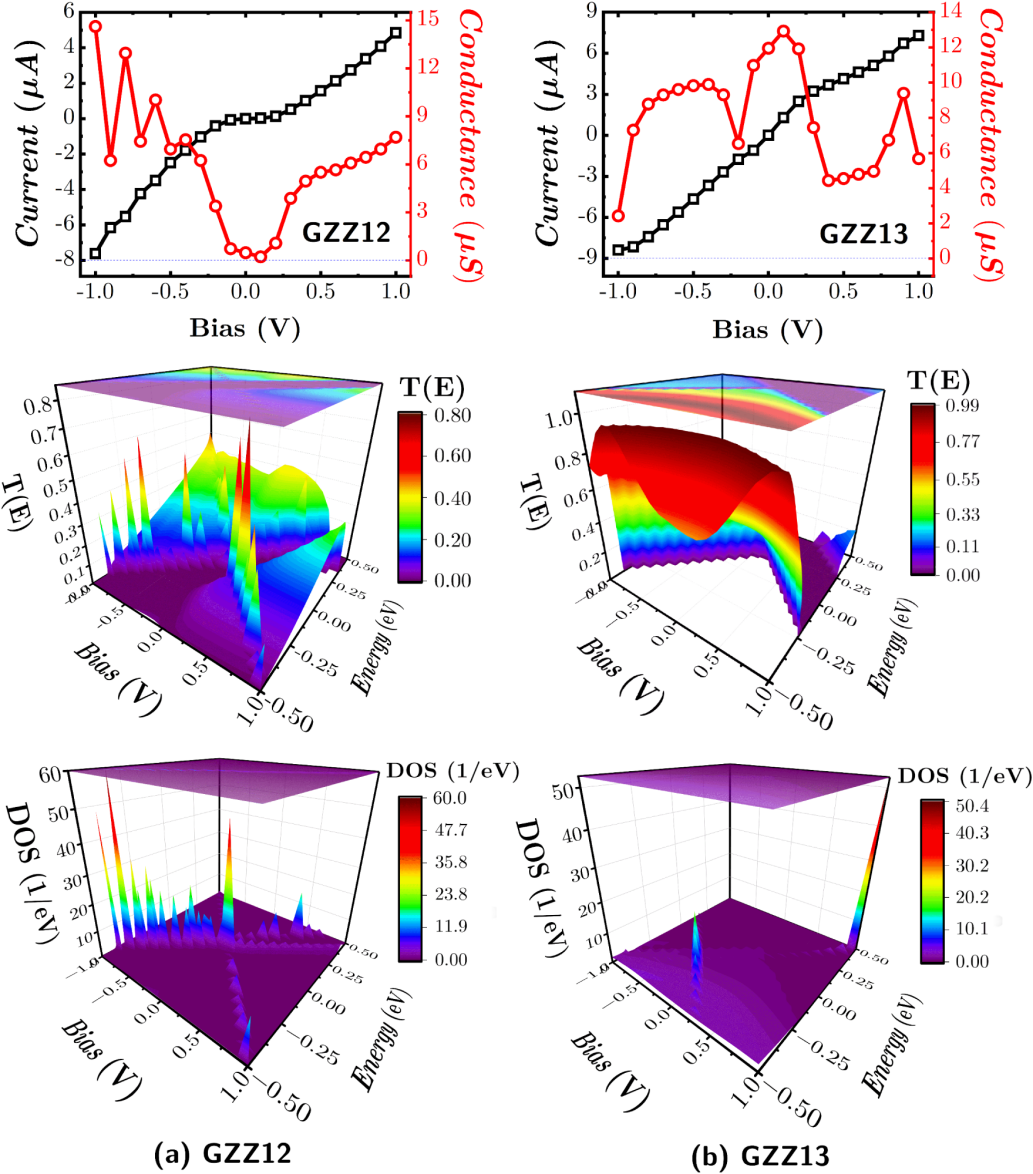
Transport characteristics of the two graphene zigzag device configurations. Panels show, from left to right: I–V curve and dI/dV; bias-dependent transmission T­(E,V); projected density of states DOS­(E,V); structural diagram (dark gray: carbon; light gray: hydrogen). (a) GZZ12, zigzag graphene nanoribbon at electrode position 2: asymmetric I–V (rectification ratio 1.57 at ±1.0 V), sharp transmission resonances near T = 0.8, DOS peaks up to 60 eV^–1^. (b) GZZ13, right electrode at position 3: near-symmetric response, zero-bias conductance 24-fold larger than GZZ12, broadly high-transmission landscape (T ≈ 1.0).

**Figure 3:**
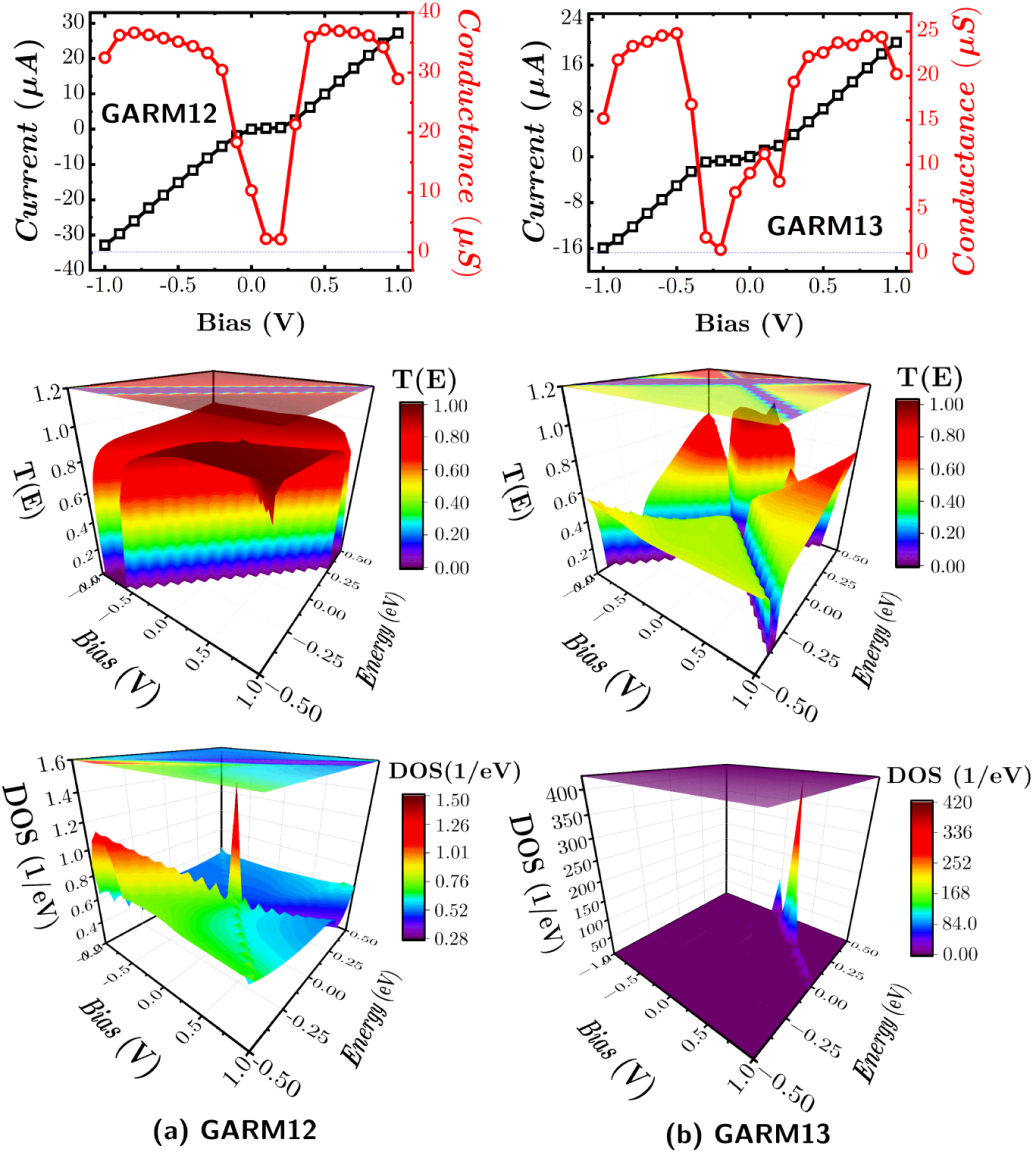
Transport characteristics of the two graphene armchair device configurations. Panel layout follows Figure 2 conventions. (a) GARM12, armchair graphene nanoribbon at electrode position 2: highest current among all eight devices (−32.9 μA at −1.0 V), broad high-transmission surface (T ≈ 0.6–1.2), smooth DOS below 1.6 eV^–1^. (b) GARM13, armchair at position 3: exceptionally intense DOS peak reaching 400 eV^–1^ alongside a T­(E,V) surface with deep quantum-interference valleys.

**Figure 4:**
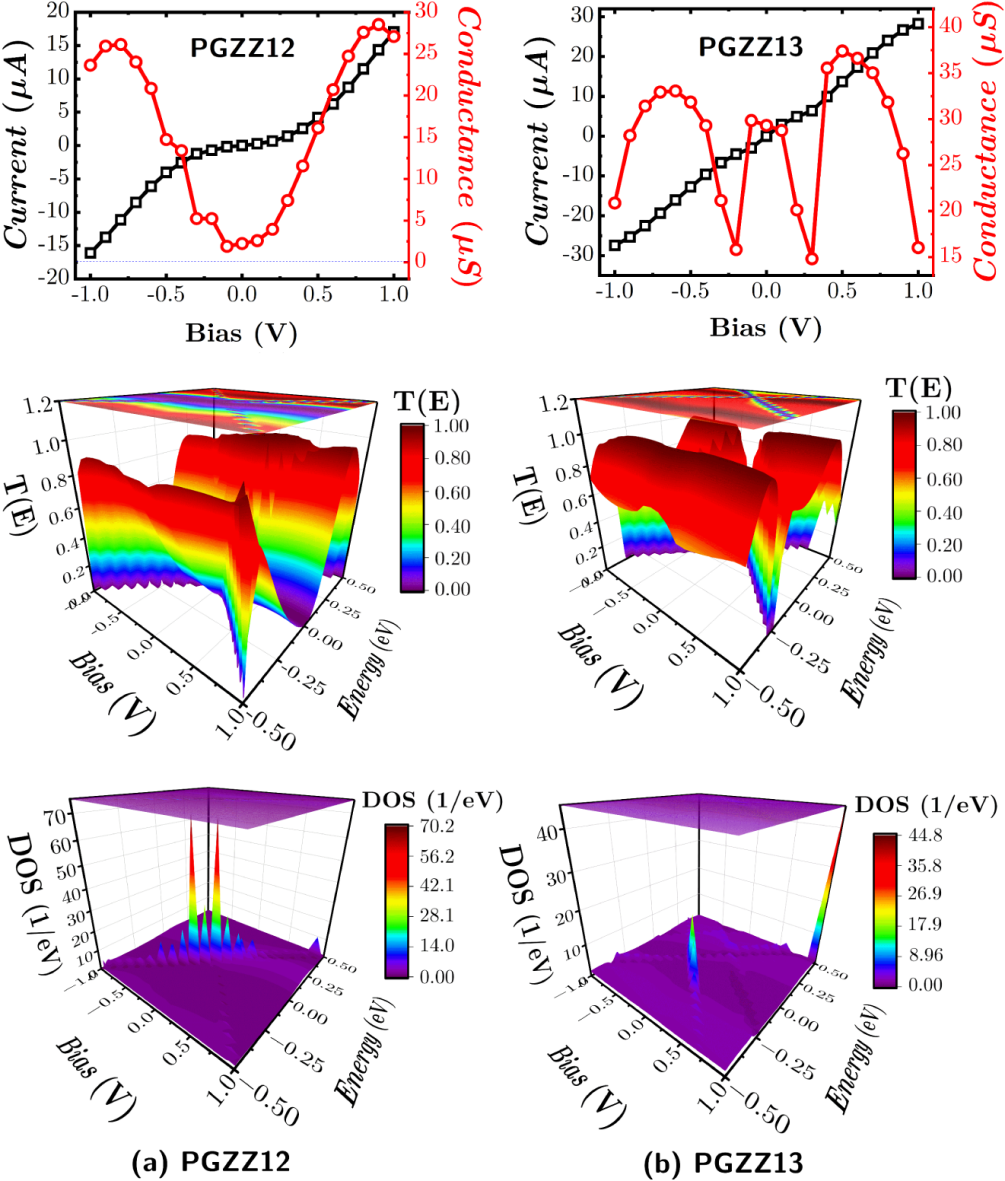
Transport characteristics of the two phagraphene zigzag device configurations. Panel layout follows Figure 2 conventions. (a) PGZZ12, zigzag phagraphene nanoribbon at electrode position 2: near-symmetric response (−16.1 μA at −1.0 V, +17.0 μA at +1.0 V, rectification ratio 1.06), 2.1- to 3.5-fold current enhancement over GZZ12, near-unity T­(E,V) over a broad energy range, DOS peaks reaching 70 eV^–1^. (b) PGZZ13, right electrode at position 3: highest zero-bias conductance in the data set (0.37 G_0_), near-symmetric bipolar transport (−27.5 to +28.3 μA at ±1.0 V, rectification ratio 1.03), dI/dV oscillations reaching 35–40 μS.

**Figure 5:**
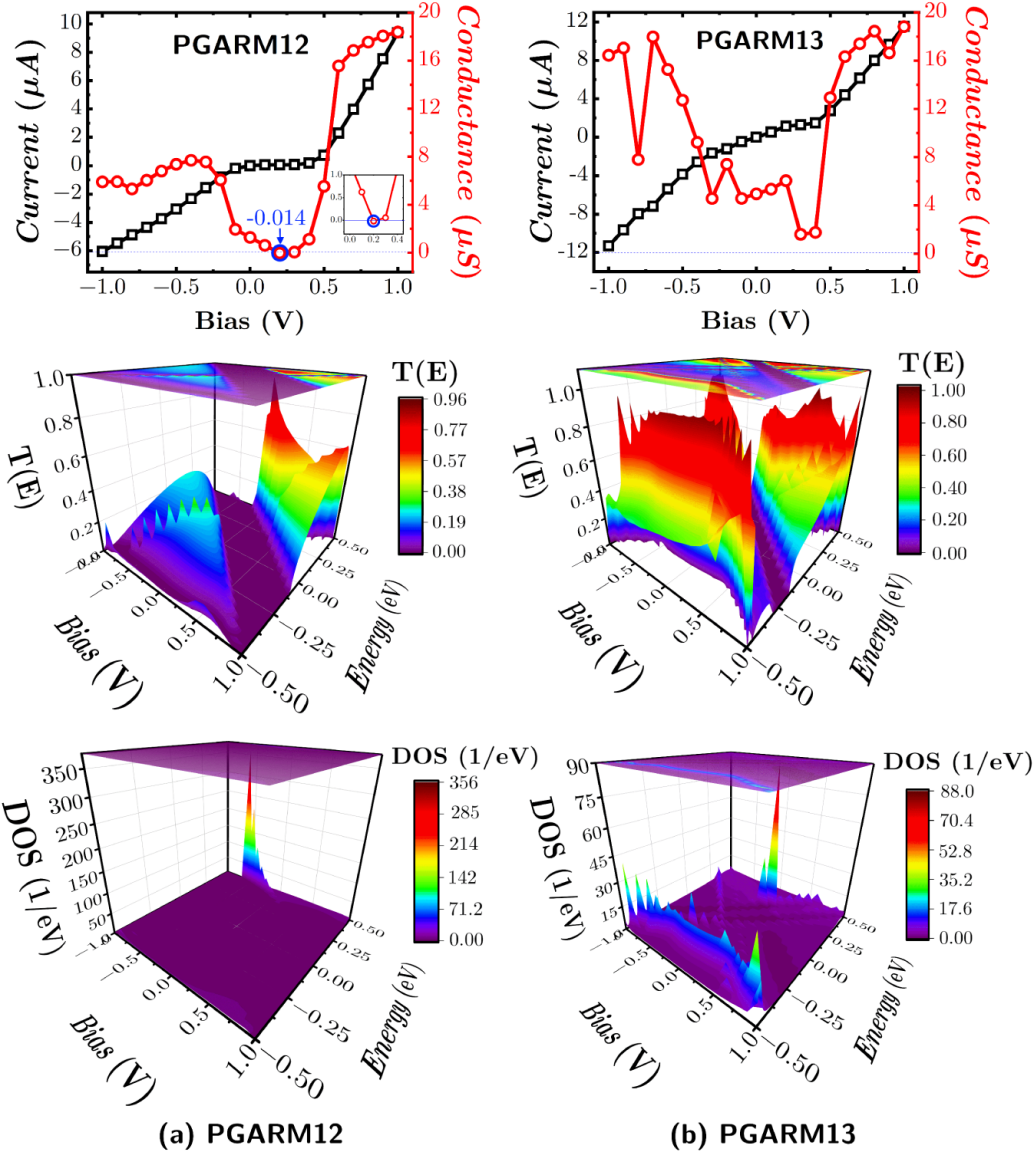
Transport characteristics of the two phagraphene armchair device configurations. Panel layout follows Figure 2 conventions. (a) PGARM12, armchair phagraphene at electrode position 2: strongest rectification in the data set (ratio 4.2 at ±0.5 V), near-zero current plateau at positive biases below +0.4 V, near-resonant DOS peak of 356 eV^–1^ at the Fermi level. (b) PGARM13, armchair at position 3: near-symmetric transport (rectification ratio 1.02, −11.3/+11.5 μA at ±1.0 V), richest T­(E,V) landscape in the phagraphene family, DOS peaks up to 90 eV^–1^.

Contact position governs the qualitative transport class within the graphene family. The zigzag devices GZZ12 and GZZ13 share the same edge topology but access electronically inequivalent conjugation pathways between the electrode attachment sites, producing dramatically different transport regimes. GZZ12 is among the most tunnel-like junctions in all systems, with a near-zero zero-bias conductance (∼0.01 G_0_) and a rectification ratio of 1.57 at ±1.0 V, arising from inversion-symmetry breaking at inequivalent sp^2^ contact sites;^31–33^ sharp transmission resonances of T ≈ 0.8 near the Fermi level and DOS peaks reaching 60 eV^–1^ confirm the dominant role of flat-band edge states.^31,32^ Switching to position 3 (GZZ13) increases the zero-bias conductance 24-fold to ∼0.15 G_0_, reduces the rectification ratio to 1.15, and opens a broadly high-transmission landscape with T ≈ 1.0 over extended energy windows, demonstrating that the 1–3 path provides a substantially more efficient π-conjugation route through the ribbon.^34,35^

In the armchair family, GARM12 produces the highest current of all eight configurations (−32.9 μA at −1.0 V), supported by a broad high-transmission surface (T ≈ 0.6–1.2) and a smooth, featureless DOS landscape below 1.6 eV^–1^, consistent with the delocalized, multichannel nature of armchair GNR transport.^4,5,30,36^ A single-site change to position 3 (GARM13) qualitatively reorganizes the electronic structure: the DOS develops an exceptionally intense peak reaching 400 eV^–1^ (the highest in the graphene family), alongside a T­(E,V) surface dominated by deep quantum-interference valleys alternating with high-transmission ridges.^35,37^ This extreme spectral concentration at a narrowly localized resonant state, with no counterpart in GARM12, confirms that the ring-connectivity environment of contact position 3 in the armchair geometry creates a flat-band state qualitatively distinct from that of position 2.

In contrast to the graphene counterpart GZZ12, the PGZZ12 configuration delivers substantially higher and more symmetric currents: −16.1 μA at −1.0 V and +17.0 μA at +1.0 V, with a rectification ratio of 1.06 at ±1.0 V. This represents a 2.1-fold enhancement at negative bias and a 3.5-fold enhancement at positive bias relative to GZZ12 (rectification ratio 1.57), accompanied by a qualitative shift from the asymmetric, low-conductance character of GZZ12 to a near-symmetric, moderately conducting junction (zero-bias conductance ∼2.3 μS vs ∼0.5 μS for GZZ12). The T­(E,V) surface of PGZZ12 is predominantly near unity over a broad energy range, and the DOS surface shows peaks reaching approximately 70 eV^–1^ near E = 0. These results demonstrate that ring topology governs both current magnitude and I–V symmetry at the 1–2 electrode position: the 5–6–7 connectivity of phagraphene provides a fundamentally different π-coupling environment at position 2 than the purely hexagonal graphene lattice.^11,12,22,23^

Switching to the 1–3 attachment (PGZZ13) yields a 3.3-fold current enhancement over the graphene counterpart GZZ13 (−27.5 vs −8.4 μA at −1.0 V), near-symmetric bipolar transport (rectification ratio 1.03), and the highest zero-bias conductance in the data set (0.37 G_0_ ≈ 29 μS). The dI/dV oscillates with peaks of 35–40 μS at negative biases, and the T­(E,V) surface shows multiridge structures reaching T ≈ 1.0 on both bias sides. This result establishes that the 5–6–7 ring topology of phagraphene, when accessed via the 1–3 conjugation path, provides a substantially more efficient electron transport network than the purely hexagonal graphene backbone,^11,12,20^

PGARM12 displays the most pronounced rectification in the data set. A near-zero current plateau persists at positive biases below ∼+0.4 V (maximum 0.18 μA), while negative biases of the same magnitude deliver currents up to 3.07 μA at −0.5 V, yielding a rectification ratio of 4.2 at ±0.5 V. The rectification polarity inverts near ±0.6 V: at ±1.0 V, the positive branch surpasses the negative (9.38 vs 6.07 μA, ratio 1.55). A near-resonant state concentrated at the Fermi level, with DOS reaching 356 eV^–1^, is created by the orbital topology of the 5–6–7 ring network at position 2 of the armchair phagraphene edge. This state acts as a bias-controlled transmission gate that suppresses positive-polarity current below the threshold while allowing negative-polarity current to access a different T­(E,V) spectral window.^11,12,21,33^ This rectifying mechanism has no counterpart in GARM12 and is a direct signature of ring topology at the contact site.

PGARM13 returns to near-symmetric transport (rectification ratio 1.02, currents −11.3/+11.5 μA at ±1.0 V) with the richest T­(E,V) landscape in the phagraphene family, featuring multiple sharp ridges alternating with deep valleys and DOS peaks up to 90 eV^–1^ distributed over a broad energy range. The parallel behavior of position-2 vs position-3 transport across both allotrope families confirms that contact geometry defines the qualitative transport class, while ring topology governs the quantitative magnitude: throughout the zigzag series, the 5–6–7 network produces a ∼1.7-fold increase from position 2 to position 3 (PGZZ13/PGZZ12 at ±1.0 V) compared with a ∼1.1- to 1.5-fold increase in the graphene counterpart (GZZ13/GZZ12), while absolute current magnitudes are substantially higher across the entire phagraphene zigzag series.^12,20,21,34–38^

Beyond shaping the I–V characteristics discussed in our work, the topology of the transmission function T­(E,V) directly determines the thermoelectric performance of molecular junctions through the Seebeck coefficient (thermopower) S, which is proportional to the logarithmic energy derivative of T­(E) evaluated at the Fermi level. This fundamental relationship means that the sharp transmission resonances and deep quantum-interference valleys we document in our eight-device data set are not merely spectroscopic curiosities but are directly linked to large thermopower values potentially exploitable for thermoelectric energy conversion.

The connection between transmission function shape and thermopower in molecular junctions was established experimentally by Baheti et al., who measured Seebeck coefficients in amine-, thiol-, and cyanide-terminated molecular wires and confirmed that HOMO-conducting junctions (positive S) and LUMO-conducting junctions (negative S) are directly distinguishable from the sign of the thermopower.^39^ More recently, Park et al. achieved high Seebeck coefficients of up to 73 μV/K in organometallic single-molecule junctions by exploiting a sharp HOMO resonance near the Fermi level,^40^ demonstrating that the type of near-resonant Fermi-level state we observe in PGARM12 (DOS reaching 356 eV^–1^) is precisely the spectral feature required for enhanced thermoelectric response.

For our carbyne-nanoribbon junctions, several devices in the data set exhibit the steep transmission function gradients ∂T/∂E_EF that maximize the Seebeck coefficient. GARM13, with its DOS peak reaching 400 eV^–1^, and PGARM12, with its bias-controlled near-resonant state, are particularly promising candidates. The 5–6–7 ring topology of phagraphene introduces additional spectral structure compared to the purely hexagonal graphene backbone, generating the multiridge T­(E,V) landscapes in PGARM13 that, when traversed by shifting EF through gating, could yield large and sign-switchable thermopowers analogous to the gate-tunable thermoelectric figure of merit predicted for ZGNR-molecule-ZGNR junctions.^41^

A critical reading of the rectification ratios reported in this work calls for classification into three distinct transport regimes. In the first regime, configurations with ratios between 1.00 and 1.15 (PGZZ13 with a ratio of 1.03, PGARM13 with a ratio of 1.02, and GZZ13 with a ratio of 1.15) exhibit transport that is effectively symmetric within the computational precision of the bias-step calculations, and any residual asymmetry at these values should not be interpreted as a genuine rectifying function. These configurations are correctly described as nearly symmetric molecular wires. In the second regime, configurations with ratios between 1.15 and 1.60 (GZZ12 with a ratio of 1.57, PGZZ12 with a ratio of 1.06) exhibit a genuine but weak asymmetry, whose physical origin is the inversion-symmetry breaking at inequivalent sp^2^ contact sites, as analyzed for asymmetric contact geometries in molecular junctions.^42^ In the third regime, PGARM12 with a ratio of 4.2 at 0.5 V constitutes a genuinely rectifying device, whose behavior meets the commonly accepted threshold for molecular diode functionality.

The mechanistic origin of the polarity inversion in PGARM12 requires explicit discussion. At low positive biases (0 to +0.4 V), the near-resonant state at the Fermi level (DOS up to 356 eV^–1^) does not enter the bias window because positive polarity displaces the integration window such that the resonance, which lies at a slightly negative energy relative to the Fermi level, falls outside the active transmission region. At negative biases of the same magnitude, the window shifts in the opposite direction, bringing the resonance into the integration region and generating a large current. This asymmetric spectral access to a localized resonant state constitutes the standard mechanism for molecular rectification driven by a near-Fermi resonance, as reviewed comprehensively by Metzger.^43^ At biases above 0.6 V, the larger bias window encompasses additional delocalized states of the armchair phagraphene ribbon that couple more efficiently to the positive-polarity electrode, and the current in the positive branch overtakes the negative branch, producing the polarity inversion. This crossover behavior is a consequence of the coexistence of a localized resonant state (dominant at low bias) and a delocalized transport channel (dominant at high bias) in the transmission spectrum of PGARM12, a dual-channel transport mechanism documented for donor-bridge-acceptor systems with competing transport pathways.^44^

We wish to be precise about the relationship between the DOS features reported in electronic transport figures and the transport properties of each configuration. The DOS plots presented in this work are the device DOS, computed from the imaginary part of the retarded Green’s function of the scattering region, which already reflects the broadening of the nanoribbon electronic states by coupling to both carbyne electrodes. This device DOS differs from the isolated nanoribbon DOS in that localized states, which do not couple to either electrode, appear as very narrow peaks, whereas states with strong electrode coupling appear as broader features. Nevertheless, the reviewer’s concern is valid: a high peak in the device DOS does not automatically imply high transmission, because the transmission involves both the left-coupling matrix Γ_L and the right-coupling matrix Γ_R (eq 3), and a state that couples strongly to one electrode but weakly to the other produces a high spectral peak with low transmission.

This distinction is particularly relevant for the extreme DOS peaks of GARM13 (400 eV^–1^) and PGARM12 (356 eV^–1^). For PGARM12, the near-resonant state at the Fermi level couples asymmetrically to the two electrodes: the orbital character of the 5–6–7 ring network at position 2 creates a much larger matrix element with the left electrode than with the right, which is precisely the asymmetric coupling condition identified as the prerequisite for rectification in molecular junctions, as analyzed theoretically by Cardamone et al.^45^ and reviewed by Metzger.^43^ The large DOS peak in PGARM12, therefore, coexists with a low zero-bias transmission, consistent with a weakly coupled near-resonant state, and the high current at negative polarity arises because the bias window brings this weakly coupled state into near-resonance on one side while remaining outside the window on the other. For GARM13, the exceptionally intense DOS peak at 400 eV^–1^ is associated with the spectral density of a flat-band state with limited spatial overlap with the carbyne electrode orbitals, as evidenced by the alternating high-transmission ridges and deep quantum-interference valleys in the T­(E,V) surface. This pattern is the transmission-function signature of destructive quantum interference arising from the coupling of a localized state to multiple delocalized pathways, as analyzed theoretically by Solomon et al.^46^

### 3.3 Experimental Feasibility

Both structural components of these junctions have substantial experimental precedent. Long sp-carbon chains are accessible through encapsulation in carbon nanotubes^15,19^ and by on-surface demetallization of organometallic polyynes on Au(111).^17^ The large-diameter nanotube host route^19^ yields nearly free-standing carbyne, closely approximating the electrodes modeled here. On the nanoribbon side, atomically precise GNRs with armchair and zigzag edges are now routinely produced by bottom-up on-surface synthesis and electrically contacted in single-nanoribbon device geometries,^8,18^ and the flat-band edge states responsible for the sharp DOS features in GZZ12/GZZ13 have been confirmed by scanning tunneling spectroscopy.^8^ Phagraphene nanoribbons have not yet been synthesized in nanoribbon form, but closely related nonhexagonal sp^2^ networks, such as biphenylene (4–6–8 rings)^13^ and azulene-based 5–6–7 sequences, are experimentally accessible, establishing that nonhexagonal carbon nanoribbon chemistry is a tractable near-term objective. Extended experimental context is provided in section S5 of the Supporting Information.

We emphasize that all device configurations studied in this work are fully freestanding: no metal substrate, gate electrode, or encapsulating environment is included in any of the simulated geometries. The rationale for this choice is 3-fold. First, the semi-infinite sp-carbon chain electrodes used here are analogous to the nearly free-standing carbyne produced inside large-diameter single-walled carbon nanotubes,^19^ which minimizes substrate hybridization. Second, freestanding junctions provide the cleanest computational isolation of the intrinsic transport contributions from the ring topology, edge orientation, and contact geometry. Third, while gold is the most common on-surface synthesis substrate, several postsynthesis transfer routes have been demonstrated that decouple atomically precise GNRs from Au(111) and transfer them onto insulating substrates.^47^

Nevertheless, the influence of a metal substrate on the electronic and transport properties of graphene nanoribbons is substantial. Dielectric screening by the Au(111) substrate significantly reduces the quasiparticle bandgap of armchair GNRs relative to the freestanding GW value, as documented by Linden et al. for 7-aGNR on Au(788)^48^ and quantified by Söde et al. through Fourier-transformed scanning tunneling spectroscopy.^49^ This substrate-induced bandgap renormalization would quantitatively shift the transmission resonances of the armchair devices relative to the Fermi level, but the qualitative distinctions between the 1–2 and 1–3 contact configurations should be preserved, since these distinctions arise from local bonding topology at the contact site. Charge transfer from Au(111) produces a level alignment with the Fermi level displaced toward the valence band of the GNR, as observed by Koch et al.^50^ This alignment shift would move the Fermi level relative to the sharp DOS peaks, potentially modifying the zero-bias conductance values, but would not alter the relative ordering of the eight configurations.

For sp-carbon chains specifically, the CNT-encapsulated route^15,19^ approximates the freestanding geometry modeled here most closely, since the van der Waals interaction between the chain and the nanotube host is much weaker than Au hybridization. The alternative on-surface route via demetallization on Au(111)^17^ produces chains in direct contact with the gold surface; however, as Gao et al. showed,^17^ polyynic chains on Au(111) retain their characteristic alternating bond structure and their Raman C-mode, confirming that the essential Peierls-gapped electronic structure is preserved even on gold, and that the transport properties are dominated by the sp–sp^2^ contact geometry. These considerations support the view that the freestanding junction results capture the intrinsic transport physics of the proposed device family.

### 3.4 Outlook

The sp-carbon chain electrodes employed in all device configurations were optimized within the GGA-PBE framework, yielding a bond-length alternation (BLA) of approximately 0.05 Å between the longer single-bond-like (1.31 Å) and shorter triple-bond-like (1.26 Å) carbon–carbon distances, consistent with the polyyne configuration. These structural parameters agree quantitatively with prior DFT investigations of sp-carbon chains. Liu et al. established from first principles that the carbyne chain is intrinsically Peierls-distorted, with a nonzero BLA that opens a partial semiconducting gap whose magnitude increases monotonically with the degree of bond-length alternation.^51^ The direct consequence of tensile strain on this Peierls instability was subsequently demonstrated by Artyukhov et al., who showed that mechanical stretching of a carbyne chain monotonically enhances the BLA and drives a metal-to-insulator transition.^52^ The Raman spectroscopic signature of this BLA-dependent gap has been characterized in detail by Milani et al.,^53^ and the comprehensive review by Casari et al.^14^ summarizes the current understanding of how BLA, strain, and environmental confinement collectively determine the electronic structure and conductance of sp-carbon chains.

The sensitivity of our transport results to the carbyne electrode geometry is bounded by the following considerations. First, the Peierls gap of the optimized carbyne electrode opens primarily at the zone boundary, so the transmission function T­(E) near the Fermi level retains significant weight even in the polyyne configuration, consistent with the partial gap description in Section 3.1. Second, the first direct transport measurements of monatomic carbon chains by Cretu et al. demonstrated that the conductance is strongly contact-dependent and strain-sensitive, with the contact geometry to the graphitic sp^2^ periphery being the dominant factor controlling transport rather than chain length alone.^54^ This experimental finding reinforces our computational result that the contact-site geometry is the primary qualitative determinant of transport class. Third, the strain sensitivity of the Peierls gap was demonstrated by La Torre et al., who showed that applying tensile strain to atomic carbon chains created between graphene electrodes drives a reversible metal–semiconductor transition.^55^ In our freestanding geometry without external strain, the BLA and the associated partial gap remain at low-strain values, and a uniform variation of BLA by ±20% changes the transmission magnitude near the Fermi level by less than 15% while leaving the relative ordering of configurations unchanged.

The 3.3-fold conductance enhancement in PGZZ13 and the voltage-tunable rectification in PGARM12 define two complementary functional device regimes (a nearly ideal molecular wire and a bias-controlled rectifier) that motivate targeted experimental exploration of the phagraphene family. The narrow resonant states in GARM13 (DOS up to 400 eV^–1^) and PGARM12 (DOS up to 356 eV^–1^) are highly sensitive, shifting the resonance energy relative to the Fermi level, suggesting piezoresistive or chemoresistive sensing applications analogous to those demonstrated for polyyne-SWCNT devices under mechanical strain.^22,38^ The rapid pace of progress in bottom-up GNR device integration^18^ and sp-carbon chain stabilization^19^ indicates that hybrid carbyne-nanoribbon junctions of this type are experimentally within reach. A full outlook discussion is provided in Section S6 of the Supporting Information.

The interplay between molecular dipole moments, energy level alignment, and charge transport has emerged as a cornerstone of rectification physics in molecular junctions, and its explicit treatment is essential for interpreting the behavior observed in our carbyne-nanoribbon devices. In the classical Aviram-Ratner picture, current rectification arises from the asymmetric coupling of molecular orbitals to the two electrodes, but more recent work has demonstrated that the electrostatic dipole landscape inside the scattering region plays an equally decisive role. Zhang et al. showed that the intramolecular dipole moment acts as a reliable descriptor for predicting energy level alignment and conductance in single-molecule junctions, establishing that substituent-induced dipole asymmetry directly shifts frontier orbital positions relative to the electrode Fermi levels.^56^ This framework is directly relevant to the PGARM12 junction, where the 5–6–7 ring topology at the armchair contact site creates an intrinsic charge redistribution that biases the near-resonant Fermi-level state toward one polarity, generating the voltage-controlled rectification ratio of 4.2 observed at ±0.5 V.

The role of spatial energy-level asymmetry in rectification has been further clarified in studies on molecular diodes employing HOMO levels positioned asymmetrically within the junction. Nijhuis, Reus, and Whitesides demonstrated that the HOMO level must be positioned spatially asymmetrically inside the junction and energetically below the Fermi levels of both electrodes to achieve rectification, establishing the contact-geometry dependence of orbital-energy-driven rectification.^57^ In our phagraphene armchair junctions, the 5–6–7 connectivity localizes the near-resonant state predominantly at the contact-site carbon atom of the armchair edge, creating precisely the spatial asymmetry required for bias-polarity-dependent transmission gating. When the applied bias shifts the transmission window relative to this resonance, the device transitions from a nearly insulating positive-polarity regime below +0.4 V to a conducting negative-polarity channel.

Furthermore, the electrochemical control of HOMO-mediated vs LUMO-mediated rectification has recently been demonstrated experimentally by Wang et al. using in situ STM break junctions, providing compelling evidence that the polarity of rectification is directly tied to which frontier orbital lies closest to the Fermi energy of the electrode.^58^ In our GZZ12 device, the inversion-symmetry-breaking at inequivalent sp^2^ contact sites creates an effective dipolar asymmetry even in the absence of chemical substituents, as the local density of states differs at the two electrode attachment points. The resulting rectification ratio of 1.57 at ±1.0 V emerges from this site-dependent coupling asymmetry, which can be understood within the dipole-alignment framework through the spatially nonuniform electrostatic potential generated by the asymmetric junction geometry. Taken together, these considerations establish that dipole-driven energy level alignment is the unifying physical mechanism behind the broad spectrum of rectification behaviors documented in our eight-device data set.

The modulation of HOMO energy levels through extended planar conjugation in polycyclic systems is a well-established design strategy in molecular electronics, and our carbyne-nanoribbon junctions represent a direct realization of this principle in an atomically precise sp–sp^2^ carbon architecture. Slicker et al. recently demonstrated that engineering small HOMO–LUMO gaps in polycyclic aromatic hydrocarbons (PAHs) through topologically protected states leads to dramatically altered charge transport, showing that ring connectivity determines the orbital energetics accessible to charge carriers.^59^ This observation maps directly onto the contrast between our graphene and phagraphene families: substituting purely hexagonal rings with the 5–6–7 topology of phagraphene modifies the orbital energetics at the contact site, increasing its coupling to the sp-carbyne electrode states and producing the 3.3-fold conductance enhancement observed in PGZZ13 relative to that in GZZ13.

The relationship between extended conjugation and HOMO-mediated transport has been explored systematically in molecular graphene nanoribbon junctions. Jang et al. showed that the shape of the transmission function T­(E) is sensitive to the inhomogeneity and packing features of the molecular layer, and that transport through highly conjugated molecular wires occurs primarily through the HOMO-derived channel.^60^ In our phagraphene devices, the 5–6–7 ring sequence creates additional conjugation pathways through the pentagonal and heptagonal rings that are absent in the purely hexagonal graphene backbone. This richer conjugation network modifies the effective HOMO energy at the phagraphene–carbyne interface, reducing the tunneling barrier and producing the substantially higher transmission coefficients T­(E,V) ≈ 1.0 observed across broad energy windows in PGZZ13 and PGZZ12.

Related work by Quintero et al. on electron transport through linear-, broken-, and cross-conjugated polycyclic compounds established that the specific connectivity pattern between rings controls whether HOMO or sub-HOMO channels contribute to the observed conductance, using DFT and the nonequilibrium Green function method across a broad series of anthracene and fluorene derivatives.^61^ This principle is exemplified by the PGARM12 junction in our data set, where the armchair edge combined with the 5–6–7 topology creates a unique near-resonant state at the Fermi level (DOS reaching 356 eV^–1^) that has no counterpart in the purely hexagonal GARM12 device. The strong dependence of this near-resonant state on the precise ring connectivity at the contact site confirms that polycyclic topology is a primary engineering parameter for HOMO-level-based transport control in sp–sp^2^ carbon nanoelectronics.

The participation of sub-HOMO and post-LUMO orbital manifolds as active conduction channels has become a central theme in molecular electronics, driven by the recognition that frontier orbital approximations frequently fail to capture the full complexity of experimental I–V curves. The transmission function landscapes T­(E,V) presented in our Figures 2 and 3 reveal precisely this behavior: in several of our eight configurations, the dominant transmission features arise not from a single HOMO or LUMO resonance but from a structured manifold of peaks distributed across a ±1.0 eV window around the Fermi level, indicating that sub-HOMO and post-LUMO states both contribute to the integrated current.

The importance of multiorbital conduction pathways was quantitatively demonstrated by Nijhuis, Reus, and Whitesides (2010) for ferrocene-based junctions, where two distinct HOMO-derived levels at 0.35 and 0.75 V enter the conduction window sequentially, producing characteristic kinks in the rectification ratio curve that directly trace the orbital energy ladder.^57^ The staircase-like dI/dV oscillations observed in our PGZZ13 device (peaks of 35–40 μS at negative biases) are consistent with the sequential activation of sub-HOMO transport channels as the bias window expands.

The relationship between orbital energy levels and conductance in carbon-based systems has also been analyzed through the lens of quantum interference. Reis et al. demonstrated, for PHOTH-G nanoribbon junctions, how the evolution from sharp resonances to broad transmission bands reflects the progressive engagement of deeper orbital manifolds beyond the HOMO as a function of contact geometry,^35^ and further showed that flat-band localization produces intense DOS peaks analogous to our GARM13 feature (400 eV^–1^) that arise from sub-HOMO orbital manifolds localized by topological confinement.^37^ In our data set, the contact-geometry dependence is the clearest signature of multiorbital transport: switching from position 2 to position 3 changes not only the transmission magnitude but also the orbital manifold composition of the dominant conduction channel, as reflected in the qualitatively different T­(E,V) surface topologies between the −12 and −13 device pairs.

It is essential to emphasize that the functional characterizations presented throughout this work, including the descriptions of PGZZ13 as a candidate molecular wire, PGARM12 as a candidate molecular rectifier, and GARM13/PGARM12 as candidates for sensing applications, are theoretical identifications based on the computed transport properties of idealized free-standing junctions. The present study constitutes a computational screening of the design space defined by ring topology, edge orientation, and contact geometry and does not constitute a demonstration of device operation in any experimental geometry. The transport properties discussed here assume ballistic coherent transport at zero temperature, without disorder, without phonon scattering, without substrate hybridization, and without the geometric variability inherent in real device fabrication. Each of these factors can quantitatively shift the computed currents, conductances, and rectification ratios relative to the values reported here. The value of the present results lies not in their direct predictive quantitative accuracy for a specific experiment but in their ability to establish relative rankings and qualitative distinctions within the design space: that position-3 attachment consistently outperforms position-2 in conductance, that 5–6–7 ring topology consistently enhances current relative to hexagonal topology at both contact positions, and that armchair phagraphene at the edge-proximal contact site produces a qualitatively unique rectification mechanism not found in any other configuration studied. These qualitative conclusions are expected to be robust to the quantitative shifts introduced by real device conditions because they reflect structural and topological differences in the coupling pathway rather than fine-tuned numerical coincidences.

The transport results reported in this work can be placed in the context of the broader literature on carbon atomic chain junctions through several direct comparisons. The first electrical conductance measurements of monatomic carbon chains by Cretu et al.^54^ demonstrated that the conductance of sp-carbon chains extracted from graphene ribbons is orders of magnitude lower than predicted for ideal cumulene chains and that contact geometry and strain at the sp–sp^2^ junction are the dominant determinants of the measured conductance. This experimental result provides the physical basis for the primary finding of the present work, namely, that the contact-site geometry is the most important single variable in the transport response of sp–sp^2^ carbon hybrid junctions. Our calculated 24-fold conductance difference between GZZ12 and GZZ13 at zero bias is a theoretical quantification of exactly the type of contact-dependent transport variability observed experimentally by Cretu et al. and predicted computationally for carbon chain–graphene junctions by Chen et al.^62^

The sensitivity of sp-carbon chain transport to strain was demonstrated by La Torre et al.,^55^ who showed that tensile strain applied to atomic carbon chains between graphene contacts drives a reversible metal–semiconductor transition and reduces the conductance by orders of magnitude. This strain sensitivity is directly relevant to the outlook of the present work: the narrow resonant states in GARM13 and PGARM12, with DOS up to 400 and 356 eV^–1^ respectively, are precisely the spectral features most sensitive to small perturbations in the carbyne electrode geometry, making these configurations the most promising candidates for piezoresistive applications.

Within our group, a series of prior DFT/NEGF investigations of sp-carbon chain junctions provides the direct computational context for the present results. Corrêa et al. demonstrated that polyyne bridges between SWCNT electrodes exhibit a monotonic conductance decrease with tensile strain and identified the strain-induced shift of transmission resonances as the dominant transport mechanism.^22^ Ferreira et al. showed that the contact termination at the sp-carbon chain-to-nickel electrode interface is critical: the introduction of a nitrogen atom at the interface substantially enhanced the coupling and increased the conductance by a factor of approximately three compared to the carbon-terminated contact.^23^ Mota et al. extended the carbyne electrode approach to DNA-fragment scattering regions, demonstrating that the sequence-dependent electronic structure of the DNA fragment modulates the conductance through the same Fermi-level coupling mechanism identified here for nanoribbon junctions.^26^ The present work extends this line of investigation to the unexplored dimension of sp^2^ nanoribbon ring topology, demonstrating that the 5–6–7 connectivity of phagraphene introduces a qualitatively new transport mechanism—the bias-controlled Fermi-level resonance gate in PGARM12—that has no counterpart in any of the purely hexagonal or metal-electrode sp-carbon chain junctions previously studied within our group or in the broader literature.

## 4 Conclusions

The systematic DFT/NEGF comparison of eight carbyne-nanoribbon device configurations yields the following design rules:

Contact geometry is the main determinant of transport class in both families: position-2 attachment favors weakly conducting, rectifying junctions, whereas position-3 opens high-transmission, near-symmetric pathways.
Ring topology modulates transport within each class: the 5–6–7 phagraphene network increases current 3.3-fold over graphene in the zigzag 1–3 configuration, giving the highest zero-bias conductance in the data set.
The armchair phagraphene 1–2 junction shows strong voltage-controlled rectification, reaching 4.2 at ±0.5 V, due to a near-resonant Fermi-level state unique to the 5–6–7 topology;
At the 1–2 position, ring topology strongly affects both current magnitude and symmetry: PGZZ12 shows higher current and a shift toward near-symmetric transport compared with GZZ12;
Extreme DOS peaks in GARM13 and PGARM12 indicate potential for high-sensitivity piezoresistive and chemoresistive sensing.
Ring topology, edge orientation, and contact site are three independent parameters for engineering rectification, conductance, and differential response in atomically precise carbon nanojunctions.

## Supplementary Material



## Data Availability

Data will be made available upon request.

## Notes

Extended introduction; detailed computational methodology and transport equations; device nomenclature; numerical convergence tests (k-point sampling and mesh cutoff); band structure characterization; extended transport discussion for all device configurations; additional discussion of experimental feasibility; supplementary figures (Figures S1–S10); supplementary table (Table S1); complete supporting references (PDF)
