# Supplementary material for: Atomically Precise Carbyne-Nanoribbon Devices: Transmission Engineering through Ring Topology and Contact-Site Selection
Source: ACS Omega. 2026 Jul 24;11(31):46866–79. doi: 10.1021/acsomega.6c05188 (PMC13470822; doi:10.1021/acsomega.6c05188)
Supplement: Supplementary file 1 [file ao6c05188_si_001.pdf]

Supporting Information

Atomically Precise Carbyne-Nanoribbon Devices: Transmission Engineering through Ring Topology and Contact-Site Selection

Ana Beatriz Rosa da Silva,[a] Iurbson Sales Costa,[b] Alessandre Sampaio-Silva,[a,*] Jordan Del Nero [b]

[a] Departamento de Física, UEPA, Belém, 66050-540, Brazil

[b] Faculdade de Física, UFPA, Belém, 66075-110, Brazil

*Corresponding Author: alessandre@uepa.br

S1. Extended Introduction

This section provides the detailed literature context including discussion of graphyne and graphdiyne, phagraphene synthesis precedents, and experimental progress on sp-carbon chain stabilization.

S1.1. Non-hexagonal Carbon Allotropes and Related Networks

Carbon's structural versatility spans hybridization states from three-dimensional sp^3 diamond to two-dimensional sp^2 graphene and one-dimensional sp carbyne, making it uniquely suited as a single-element platform for atomically precise nanoelectronics [1–3]. The discovery of graphene by Novoselov et al. in 2004 marked a landmark achievement in materials science, demonstrating that a single atomic layer of carbon atoms arranged in a honeycomb hexagonal lattice could be mechanically exfoliated from graphite and electrically characterized [1]. This discovery revealed that two-dimensional (2D) materials can exhibit extraordinary electronic, mechanical, and thermal properties that are inaccessible in their bulk counterparts [2,3]. Graphene is characterized by a linear dispersion relation near the Dirac points, resulting in charge carriers that behave as massless Dirac fermions at room temperature [2].

When graphene is patterned or grown into narrow strips, it forms graphene nanoribbons (GNRs), quasi-one-dimensional (1D) structures in which quantum confinement and edge geometry profoundly determine the electronic character [4,5]. Armchair-edged GNRs display a width-dependent energy bandgap spanning from near zero to values exceeding one electron volt [4,5]. Zigzag-edged GNRs host localized edge states near the Fermi level associated with magnetic ordering along the ribbon edges [4]. The first experimental confirmation of energy gap engineering in lithographically defined GNRs was provided by Han et al. [5]. Baringhaus et al. subsequently reported ballistic electron transport in epitaxially grown GNRs over distances exceeding 10 μm at room temperature [6].

Bottom-up on-surface synthesis has emerged as the most successful strategy for achieving atomic-level precision in nanoribbon fabrication [7,8]. Cai et al. reported the first bottom-up synthesis of GNRs with atomically precise armchair edges from polycyclic aromatic hydrocarbon precursors on gold surfaces [7]. Ruffieux et al. extended this methodology to GNRs with zigzag edge topology, confirming by scanning tunneling spectroscopy the presence of edge-localized electronic states [8]. Narita et al. reviewed progress in bottom-up synthesis and highlighted the richness of the structural space accessible through rational molecular design [9]. Rizzo et al. fabricated topologically nontrivial GNRs hosting in-gap states at their termini [10].

A particularly significant finding from on-surface synthesis is that nanoribbon carbon backbones produced under certain conditions contain non-hexagonal rings, arising from Stone-Wales rearrangements during annealing. Koskinen et al. demonstrated theoretically that zigzag edges of graphene undergo spontaneous self-passivating reconstructions generating pentagon-heptagon ring pairs [S4]. Lahiri et al. reported the experimental realization of a linear extended defect in graphene composed of alternating pentagons and octagons acting as a metallic quantum wire [S1]. Fan et al. reported the on-surface synthesis of a nonbenzenoid carbon allotrope network built from biphenylene units, yielding a periodic lattice of four-membered, six-membered, and eight-membered carbon rings with metallic character confirmed by scanning tunneling spectroscopy [13].

The interest in structurally diverse carbon allotropes extends to networks incorporating sp-hybridized atoms within 2D layers. Graphyne and graphdiyne are 2D allotropes in which some C-C bonds of the hexagonal lattice are replaced by acetylenic or diacetylenic linkages [S2,S3]. Baughman et al. provided the first systematic theoretical analysis of graphyne-family networks, predicting stable planar carbon forms with tunable band structures and identifying specific configurations with Dirac-cone-like dispersions [S2]. The experimental realization of graphdiyne was achieved by Li et al., who synthesized large-area thin films by cross-coupling reactions on copper foil, demonstrating p-type semiconducting behavior [S3]. Phagraphene, a 2D carbon network composed entirely of sp^2-hybridized atoms arranged in alternating pentagonal, hexagonal, and heptagonal rings, was predicted through crystal structure searches by Wang et al., who found that its total energy lies close to that of graphene and that it supports distorted Dirac cones at the Fermi surface [11].

S1.2. Carbyne: Synthesis, Stability, and Transport

Among all carbon allotropes, carbyne occupies a unique position as the one-dimensional form in which every carbon atom is sp-hybridized, producing a linear chain with either alternating single and triple bonds in the polyyne form or cumulated double bonds in the cumulene form [14]. The properties predicted for the infinite carbyne chain include extraordinary mechanical stiffness exceeding that of all other known materials and a bandgap tunable from metallic to wide-gap semiconducting [14]. Casari et al. provided a comprehensive review of sp-carbon chains, covering their spectroscopic characterization, synthesis in solution and the gas phase, and key open questions [14]. Shi et al. demonstrated that chains of sp-hybridized carbon atoms can be stabilized

at room temperature by confinement within double-walled carbon nanotubes [15]. Chalifoux and Tykwinski synthesized polyynes containing up to 44 consecutive sp-hybridized carbon atoms [16]. The electronic transport properties of finite sp-carbon chains bridging nanoscale electrodes are strongly influenced by chain length, contact geometry, and external mechanical perturbations. Correa et al. investigated the conductance of polyyne bridges connected to SWCNT electrodes under mechanical strain [22]. Ferreira et al. examined the transport in sp-carbon wires coupled to atomic-size nickel electrodes [23]. Mota et al. explored hybrid devices in which sp-carbon chains serve as electrodes flanking a DNA fragment [26].

S1.3. Electronic Transport in Phagraphene-Based Structures

The electronic transport properties of phagraphene-derived structures have been investigated with increasing depth. Da Silva Jr. et al. demonstrated that zigzag phagraphene nanoribbons undergo a topological insulator-to-metal transition as a function of ribbon width [12]. Nisioka et al. showed that boron or nitrogen substitution in zigzag phagraphene nanoribbons systematically modifies the charge transport characteristics [20]. Santos et al. examined the electrical behavior of heterojunctions built by connecting graphene and phagraphene segments in series, finding transitions ranging from metallic conduction through tunneling to rectifying behavior [21]. Sampaio-Silva et al. extended the analysis to include inelastic scattering effects in doped phagraphene devices [38]. These results, summarized in Table S1, establish zigzag phagraphene nanoribbons as electronically versatile nanostructures highly sensitive to structural and chemical perturbations.

Table S1. Selected electrical properties reported in the literature (E = experimental, T = theoretical / computational) for carbon allotrope nanoribbons and molecular junctions incorporating sp-hybridized carbon chain segments. G_0 = quantum of conductance ($2e^2/h$). NDR = negative differential resistance; IETS = inelastic electron tunneling spectroscopy.

System / Nanoribbon	Ring Types / Hybridization	Electronic Character	Bandgap / Transport Notes	Type	Ref.
Graphyne nanoribbon (α, β, γ)	Hexagonal + acetylenic / sp + sp^2	Metallic to semiconducting; Dirac-cone dispersions in α-graphyne	0 to ~0.8 eV (theoretical)	T	[S2]
Graphdiyne nanoribbon	Hexagonal + diacetylenic / sp + sp^2	p-type semiconductor; direct bandgap	~0.46 eV (DFT); p-type confirmed in experiment	E/T	[S3]

Zigzag phagraphene nanoribbon (ZPGNR)	5-6-7 / sp^2	Topological insulator-to-metal transition vs. width	0 to ~0.3 eV; 0 to ~1 G_0 (DFT/NEGF)	T	[12]
B,N-doped ZPGNR	5-6-7 / sp^2 + dopants	Semiconductor with tunable transport gap	Dopant-dependent modification of gap and conductance	T	[20]
Graphene-phagraphene-graphene junction	6-6-6 + 5-6-7 / sp^2	Metallic, tunneling, or rectifying	Rectification and NDR features (DFT/NEGF)	T	[21]
Doped phagraphene device (inelastic)	5-6-7 / sp^2 + dopant	Semiconductor with phonon-modulated transport	Electron-phonon signatures in IETS; conductance modified	T	[36]
Polyyne bridge / SWCNT junction	Linear sp + sp^2 (SWCNT)	Molecular junction; strain-sensitive conductance	Monotonic conductance decrease under tensile strain (DFT/NEGF)	T	[22]
sp-Carbon wire / Ni electrode	Linear sp + Ni metal	Conductance dominated by contact geometry	Critically contact-dependent (DFT/NEGF)	T	[23]
Carbyne-DNA-Carbyne device	sp + mixed (DNA)	Molecular junction; sequence-tunable conductance	sp-carbon electrodes provide atomically defined contacts	T	[30]

Extended defect (558) in graphene	5-5-8 / sp^2 (non-hexagonal)	Metallic 1D wire in 2D graphene	Zero bandgap; metallic transport (STM/STS + DFT)	E	[S1]
Biphenylene network (4-6-8 rings)	4-6-8 / sp^2	Metallic; absence of full aromatic gap	Zero bandgap; metallic by STS (on-surface synthesis)	E	[13]

S2. Full Methodology: Equations and Computational Details

The electronic structure calculations reported in this work were carried out within the density functional theory (DFT) framework as implemented in the SIESTA code [27]. The exchange-correlation energy was treated using the generalized gradient approximation (GGA) in the parametrization of Perdew, Burke, and Ernzerhof (PBE) [28], which provides a reliable description of structural and electronic properties for both sp^2- and sp-hybridized carbon systems. Core electrons were represented by norm-conserving Troullier-Martins pseudopotentials [29] in the Kleinman-Bylander separable form, while valence electron wave functions were expanded in a numerical atomic-orbital basis set of double-zeta polarized (DZP) quality for all atomic species. A real-space grid energy cutoff of 200 Ry was employed for the numerical integration of the charge density and all Hamiltonian matrix elements. An electronic temperature of 300 K was applied through Fermi-Dirac smearing throughout all self-consistent field (SCF) calculations. Geometry optimizations of the central scattering region were performed prior to each transport calculation, with convergence criteria set to a maximum residual force of 0.02 eV/Å on every atom.

Quantum transport calculations were performed within the Landauer-Büttiker formalism as implemented in TranSIESTA [30], which couples a NEGF treatment of the open-boundary device with a self-consistent DFT description of the electron density under finite applied bias. In this two-probe framework, the total device is partitioned into three regions: the semi-infinite left electrode, the semi-infinite right electrode, and a finite central scattering region.

The retarded Green's function of the scattering region at energy E and applied bias voltage V is given by

$$G^r(E, V) = [(E + i\eta)S_C - H_C(V) - \Sigma_L^r(E,V) - \Sigma_R^r(E,V)]^{-1} \qquad (1)$$

where $H_C(V)$ is the Kohn-Sham Hamiltonian of the scattering region evaluated self-consistently under the applied bias, S_C is the overlap matrix of the central region, η is a positive infinitesimal enforcing causality, and $\Sigma_{L,R}^r(E, V)$ are the retarded self-energies of the left and right carbyne electrodes encoding their hybridization with the finite scattering region. The self-energies are

evaluated iteratively via the principal-layer decimation technique applied to the surface Green's function of each semi-infinite carbyne lead [29]. The broadening matrices, which represent the rate of electron escape from the scattering region into each electrode and carry complete information about the electrode-device coupling strength, are defined as

$$\Gamma_{L,R}(E, V) = i\,[\Sigma_{L,R}^{r}(E,V) - \Sigma_{L,R}^{a}(E,V)] \qquad (2)$$

where $\Sigma_{L,R}^{a} = [\Sigma_{L,R}^{r}]^{\dagger}$ is the advanced self-energy, obtained as the conjugate transpose of the retarded self-energy. The energy- and bias-dependent transmission function, giving the probability of coherent electron transmission from the left to the right electrode at energy E and bias V, is obtained from the Fisher-Lee relation as

$$T(E, V) = \mathrm{Tr}\,[\Gamma_{L}(E,V)\, G^{r}(E,V)\, \Gamma_{R}(E,V)\, G^{a}(E,V)] \qquad (3)$$

where $G^{a}(E, V) = [G^{r}(E, V)]^{\dagger}$ is the advanced Green's function. The electrical current through the device is then calculated using the Landauer-Büttiker formula

$$I(V) = -(2e/h) \int_{-\infty}^{+\infty} T(E, V)\, [f_{L}(E,V) - f_{R}(E,V)]\, dE \qquad (4)$$

where e is the elementary charge, h is Planck's constant, and $f_{L,R}(E, V) = \{1 + \exp[(E - \mu_{L,R})/k_{B}T]\}^{-1}$ are the Fermi-Dirac distribution functions of the left and right electrodes, with electrochemical potentials $\mu_{L} = E_{F} + eV/2$ and $\mu_{R} = E_{F} - eV/2$, where k_{B} is the Boltzmann constant and T is the temperature. The bias voltage imposes an electrochemical potential difference eV between the two leads, defining an effective integration window $[\mu_{R}, \mu_{L}] = [E_{F} - eV/2, E_{F} + eV/2]$ within which the transmission function is actively sampled by the Fermi-function difference, so that only spectral features of $T(E, V)$ falling within or near this window contribute significantly to the total current.

Regarding electrode and scattering-region partitioning: the total device is divided into three non-overlapping regions. The two semi-infinite sp-carbyne chain electrodes constitute the left and right leads, and their surface Green's functions are computed iteratively via the principal-layer decimation technique applied to the bulk electrode unit cell. The finite central scattering region contains the nanoribbon together with several repeat units of the carbyne chain on each side, ensuring that the electron density in the buffer zone converges to the bulk electrode reference before the boundaries are imposed. This partitioning follows the standard two-probe framework of TranSIESTA [30].

Regarding geometry optimization under bias: the atomic positions of the scattering region were optimized at zero bias prior to the transport calculations, with a maximum residual force criterion of 0.02 eV/Å on every atom, as stated in Section S2 of the Supporting Information. Geometry relaxation was not repeated at each finite bias step; instead, the zero-bias optimized geometry was used throughout the I-V scan. This is consistent with standard practice in DFT/NEGF studies of rigid covalently bonded carbon junctions, where structural changes under bias are expected to be negligible compared with the electronic redistribution [30,34,36].

Regarding boundary conditions and dipole corrections: the TranSIESTA implementation enforces periodic boundary conditions in the transverse direction and open boundary conditions along the transport axis via the electrode self-energies. The electrochemical potentials of the left and right leads are set symmetrically as $\mu^L = E^M + eV/2$ and $\mu^R = E^M - eV/2$, so that the applied bias drops symmetrically across the junction with the Fermi level of the unbiased system as the reference (equation 4 in Section S2 of the Supporting Information). Dipole corrections were not applied because the electrodes are purely sp-carbon chains without a permanent dipole moment and the device architecture is symmetric with respect to the plane midway between the two leads; no net dipole accumulation is expected at the electrode-nanoribbon interface under these conditions [30].

Regarding the distribution of the bias drop across the junction: within the self-consistent NEGF-DFT cycle, the Hartree potential of the scattering region is updated at each bias voltage to reflect the charge redistribution driven by the applied bias. The potential drop is therefore determined self-consistently rather than imposed as a fixed profile. For the carbyne-nanoribbon junctions studied here, the potential drop is expected to be distributed across the electrode-nanoribbon contact regions, consistent with the results reported for related sp-carbon chain molecular junctions [22,23]. The complete set of equations governing the self-consistent transport calculation, including the retarded Green's function, the electrode broadening matrices, and the Landauer-Büttiker current formula.

The label GZZ12 designates the device in which a zigzag-edged graphene nanoribbon constitutes the scattering region and the left and right carbyne electrodes are connected at positions 1 and 2, respectively. GZZ13 identifies the same zigzag graphene geometry with the right electrode at position 3. GARM12 and GARM13 refer to the armchair-edged graphene nanoribbon devices with right electrode connections at positions 2 and 3. For the phagraphene-based junctions, PGZZ12 and PGZZ13 denote the zigzag phagraphene configurations with electrode connection pairs 1-2 and 1-3, while PGARM12 and PGARM13 identify the armchair phagraphene devices with right electrode connections at positions 2 and 3, respectively. These eight configurations collectively span the full combinatorial space of edge topology and electrode attachment geometry investigated here.

Brillouin zone integrations for the periodic electrode regions were performed using a Monkhorst-Pack k-point grid [S5] of 1×1×100 along the transport direction, which was verified to yield fully converged transmission functions for the one-dimensional sp-hybridized carbyne chain electrodes employed in all device configurations. Convergence with respect to transverse k-point sampling was also assessed, and a 1×15×1 transverse grid was found sufficient to produce transmission spectra indistinguishable from denser grids across all eight device configurations. The I-V characteristics were computed for bias voltages from 0 to +/- 1.0 V in 0.1 V steps, and the differential conductance dI/dV. All finite-bias self-consistent transport calculations were converged with the charge density updated at each bias step [30,37]. The complete computational

protocol is fully consistent with the methodology validated in prior DFT/NEGF investigations of carbon-based nanoribbon devices and sp-carbon chain molecular junctions from our group [34–37].

All calculations reported in this work were performed within the spin-unpolarized (non-magnetic) framework. This choice is fully justified by the structural details of our device configurations. In all eight junction geometries studied here, every dangling bond along the nanoribbon edges is passivated by a single hydrogen atom, as shown in the structural diagrams of Figure 1. This full single-hydrogen edge passivation is the critical factor that determines whether magnetic ordering can be sustained. The magnetic properties of zigzag graphene nanoribbons depend sensitively on the degree and pattern of edge hydrogenation: in the idealized single-hydrogen monopassivated configuration, the antiferromagnetic ground state with opposite spin polarization on the two edges is stabilized by the sublattice imbalance inherent to the zigzag termination [31,32]. However, this magnetically ordered state is strongly suppressed once the edge carbon atoms are saturated by a complete single-hydrogen capping that quenches the reactive dangling bonds.

The key theoretical and experimental evidence supporting the spin-unpolarized treatment for our systems is as follows. Ruffieux et al. [8] demonstrated by scanning tunneling spectroscopy that atomically precise zigzag GNRs fabricated by on-surface synthesis and fully passivated with hydrogen exhibit edge states whose magnetic character is strongly reduced relative to the bare-edge prediction, because the chemical reactivity of the spin-polarized edge states drives spontaneous saturation under ambient conditions. Furthermore, Magda et al. [S6] reported that magnetic ordering of zigzag GNRs is observable only in exceptionally narrow ribbons under low-temperature, ultra-clean conditions and is absent in hydrogen-passivated samples measured at room temperature. The theoretical foundation for the intrinsic magnetic instability of the bare zigzag edge was established by Pisani et al. [S7], who showed within spin-unrestricted DFT that zigzag-edge ribbons are intrinsically unstable toward magnetic polarisation — an instability that is quenched in our systems by the complete single-hydrogen passivation of all edge dangling bonds, as evidenced by the room-temperature measurements of Magda et al. cited above.

Moreover, the phagraphene zigzag nanoribbons (PGZZ12 and PGZZ13) contain fully hydrogen-passivated edges at the 5-6-7 ring boundaries. The 5-6-7 connectivity introduces bond-length alternation and ring curvature at the zigzag edge that further reduces the degree of sublattice imbalance compared to the purely hexagonal zigzag edge, making the suppression of edge magnetism even more complete than in graphene [12]. For these reasons, the spin-unpolarized treatment is the physically appropriate level of theory for all eight configurations studied here, and the transport results are not expected to be qualitatively altered by spin polarization effects in any of the geometries considered.

We agree that the quantitative current-enhancement factors reported in the manuscript require explicit numerical justification. We have therefore added Numerical Convergence Tests to

the Supporting Information, with two new figures (Figures S1 and S2), reporting systematic convergence checks of the total energy with respect to k-point sampling along the transport direction and real-space mesh cutoff, performed independently for the graphene-based and phagraphene-based device families.

Figure S1 shows the total energy of the zigzag and armchair graphene devices (GZZ and GARM, panels a-b and c-d, respectively) as a function of the k-point sampling along the transport direction kz and of the real-space mesh cutoff. The energy varies sharply for kz below approximately 20 points and then flattens to a constant value that is reproduced to within 0.001 eV up to kz = 200, confirming that the 1x1x100 transport-direction k-mesh used throughout the production calculations lies well inside the converged regime. The mesh cutoff scan shows a pronounced transient near 100 to 140 Ry, after which the total energy decreases monotonically and asymptotically approaches a stable value by 300 to 350 Ry, with a residual variation of less than 0.05 eV between 300 and 500 Ry. This confirms that the 300 Ry mesh cutoff adopted for all production runs is sufficient to converge the total energy and, by extension, the self-consistent Hamiltonian entering the transport calculation.

Figure S2 reports the equivalent convergence tests for the zigzag and armchair phagraphene devices (PGZZ and PGARM, panels a-b and c-d, respectively), following the same protocol. The kz scan again saturates by approximately 20 points, with the total energy stable to within 0.001 eV out to kz = 200, and the mesh cutoff scan shows the same qualitative behavior as the graphene family, with a transient near 100 to 140 Ry followed by asymptotic convergence beyond 300 Ry. The close similarity between the graphene and phagraphene convergence profiles confirms that the numerical settings used in this work provide a consistent level of accuracy across both ring topologies and all eight device configurations, supporting the validity of direct quantitative comparisons between them, including the 3.3-fold and 2.1- to 3.5-fold current enhancement factors discussed in the main text.

Regarding the additional parameters raised by the referee, the electrode length included in the central region and the vacuum separation were both set following the protocol validated in our prior DFT/NEGF studies of sp-carbon chain molecular junctions [22,23,26], in which the same electrode-extension and vacuum-padding criteria were shown to yield bias-independent self-energies and negligible spurious interaction between periodic images. The double-zeta polarized basis set used for all atomic species in this work is the same basis quality employed in these previous studies and in our related carbon-nanoribbon transport investigations [34-37], where it was independently validated against transmission and current convergence. Taken together with the new k-point and mesh cutoff tests now reported in Section S8, these results confirm that the numerical protocol underlying this work fully supports the relative current comparisons on which the conclusions of the manuscript are based. We have added a sentence to Section 2 (Methodology) of the main text pointing the reader to this new convergence section.

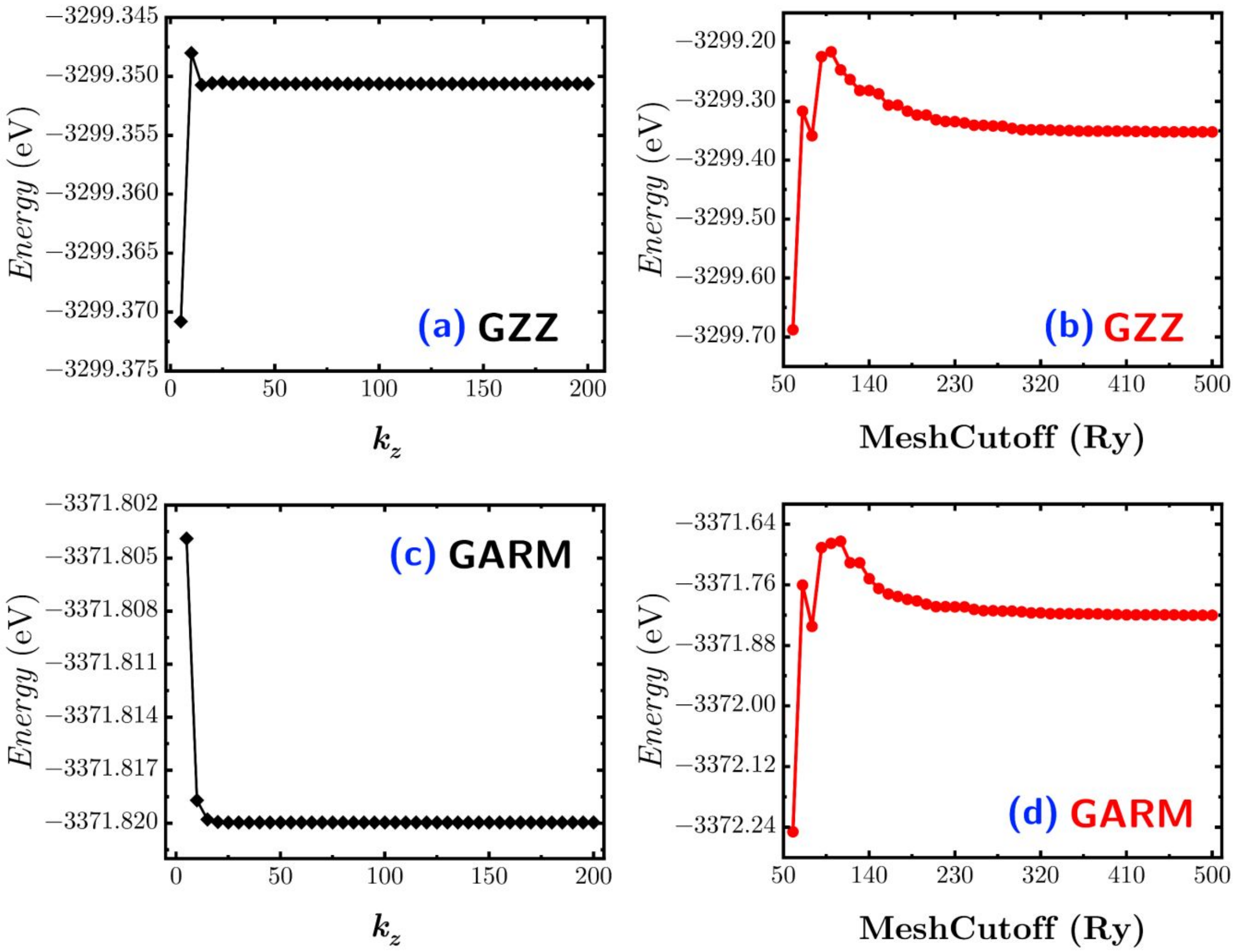

Figure S1. Convergence of the total energy with respect to k-point sampling along the transport direction kz (panels a, c) and real-space mesh cutoff (panels b, d) for the zigzag graphene device GZZ (a, b) and the armchair graphene device GARM (c, d). The total energy is converged to within 0.001 eV by kz = 20 and remains stable out to kz = 200, confirming that the 1x1x100 transport-direction k-mesh used in all production calculations lies well inside the converged regime. The mesh cutoff scan shows a transient near 100-140 Ry followed by monotonic, asymptotic convergence beyond 300 Ry, with a residual variation below 0.05 eV between 300 and 500 Ry, confirming that the 300 Ry mesh cutoff adopted throughout this work is sufficient to converge the self-consistent Hamiltonian.

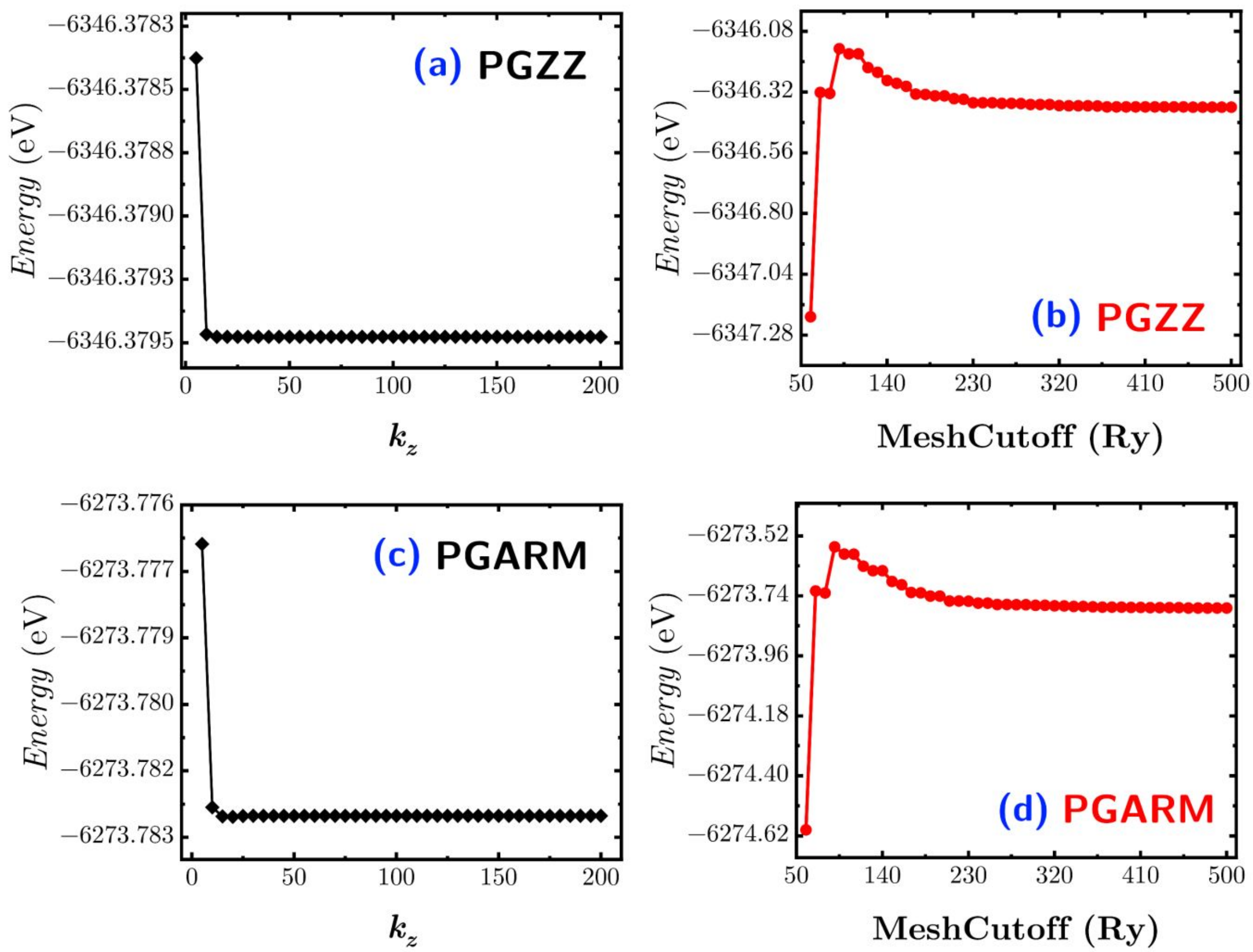

Figure S2. Convergence of the total energy with respect to k-point sampling along the transport direction kz (panels a, c) and real-space mesh cutoff (panels b, d) for the zigzag phagraphene device PGZZ (a, b) and the armchair phagraphene device PGARM (c, d). As in Figure S1, the total energy is converged to within 0.001 eV by kz = 20 and remains stable out to kz = 200, and the mesh cutoff scan shows the same transient near 100-140 Ry followed by asymptotic convergence beyond 300 Ry. The close similarity between the graphene and phagraphene convergence profiles confirms a consistent level of numerical accuracy across both ring topologies and all eight device configurations.

S3. Band Structure Characterization

Graphene constitutes the electronic reference against which all derived carbon allotropes in this work are benchmarked. Its two-dimensional honeycomb lattice of sp^2-hybridized carbon atoms hosts π and π* bands that form a linear dispersion at the inequivalent K and K' points at the corners of the hexagonal Brillouin zone, generating the Dirac cone responsible for the semimetallic character and massless-fermion behavior of charge carriers [1,2]. The zero bandgap of pristine graphene is protected by the combination of time-reversal symmetry and the sublattice symmetry of the bipartite honeycomb lattice, and any structural perturbation that breaks this symmetry generically opens a gap in the energy spectrum [1–3].

Confinement of graphene into nanoribbon geometries breaks translational symmetry in the transverse direction and introduces a new energy scale set by the ribbon width, with consequences for the band structure that depend qualitatively on the edge termination [4,5,31,32]. For armchair-edged graphene nanoribbons, quantum confinement quantizes the transverse momentum and the resulting band structure consists of one-dimensional subbands whose gap opens or closes depending on whether the allowed transverse momentum values include or exclude the Dirac point, producing three families of gap behavior as a function of ribbon width [4,31]. For zigzag-edged graphene nanoribbons, the situation is qualitatively different: the edge geometry supports a set of nearly-flat bands at energies close to the Fermi level, corresponding to states that are exponentially localized at the ribbon edges and decay algebraically into the bulk [31,32]. These localized edge states give rise to a sharp peak in the density of states near E = 0, which is one of the most distinctive band-structure signatures of zigzag graphene nanoribbons and has direct consequences for their transport properties [32].

Phagraphene introduces a fundamentally different connectivity by replacing the purely hexagonal tiling of graphene with an alternating arrangement of pentagonal, hexagonal, and heptagonal sp^2-hybridized carbon rings throughout the two-dimensional lattice [11]. The band structure of the phagraphene monolayer was predicted to host distorted Dirac cones near the Fermi level, with the distortion arising from the broken six-fold rotational symmetry of the 5-6-7 ring network relative to the honeycomb lattice [11]. When phagraphene is patterned into nanoribbons, the edge orientation again profoundly modifies the electronic character, but the interplay between the non-hexagonal ring topology and the edge geometry generates a richer phase diagram than in graphene nanoribbons. Zigzag phagraphene nanoribbons undergo a topological insulator-to-metal transition as a function of ribbon width and exhibit width-dependent transport transitions that have no counterpart in graphene [12]. Armchair phagraphene nanoribbons display a family structure in their gap behavior that also reflects the 5-6-7 ring composition, and their carrier mobility and electronic phase can be further tuned by edge chemistry and substitutional doping [20].

The sp-hybridized carbyne chains employed as electrodes in all device configurations present their own distinct band structure, which governs the coupling between the one-dimensional lead and the two-dimensional nanoribbon scattering region [14–16]. In its ideal cumulene form with cumulated double bonds, carbyne is metallic, and its band structure consists of two degenerate π bands that cross at the Fermi level, producing a fully open transmission channel at E = 0 [14]. In the polyyne form characterized by alternating single and triple bonds, bond-length alternation opens a Peierls gap that converts the chain into a semiconductor, with a gap magnitude that decreases as the degree of alternation is reduced [14,16]. In the present calculations the carbyne electrodes were modeled as geometrically optimized sp-carbon chains [28], yielding chains with slight bond-length alternation and a partially open gap, consistent with prior transport studies of sp-carbon chain junctions [22,23,26]. The coupling strength and phase relationship at the electrode-nanoribbon contact interface are determined by the local bonding geometry of the contact atom in the nanoribbon, which differs between positions 2 and 3 and between the graphene

and phagraphene ring topologies, establishing the structural basis for the transport anisotropy documented in Section 3.2 of the main text.

S4. Extended Transport Discussion

This section provides device-by-device detailed discussion of the transport characteristics, supplementing the assertive summary presented in Section 3.2 of the main manuscript.

S4.1. GZZ12

The GZZ12 device displays a markedly asymmetric I-V characteristic. At a bias of −1.0 V the device carries a current of −7.62 μA, whereas at +1.0 V the current reaches only +4.85 μA, yielding a rectification ratio of approximately 1.57 at the maximum bias explored. The asymmetry is more pronounced at intermediate voltages: at ±0.5 V the current is −2.49 μA at negative polarity and only +1.56 μA at positive polarity, giving a rectification ratio of approximately 1.59. The differential conductance dI/dV oscillates as a function of bias and shows a local maximum in the vicinity of −0.4 to −0.5 V, while on the positive side the conductance is consistently lower, confirming preferential carrier injection at the negative polarity. The T(E,V) surface of GZZ12 exhibits sharp transmission peaks of magnitude T ≈ 0.8 clustered near the Fermi energy at zero and low biases, consistent with the quasi-localized π states supported by the zigzag edge of the graphene nanoribbon [31,32]. These sharp resonances in T(E) are echoed by the DOS surface, which shows extremely localized density-of-states peaks reaching values of up to 60 eV^{-1} near E = 0, a spectral signature characteristic of the flat-band edge states known to arise in tight-binding models of zigzag graphene nanoribbons [31,32].

S4.2. GZZ13

The GZZ13 device shows a substantially more symmetric I-V around zero bias than GZZ12: at −1.0 V the current is −8.40 μA, while at +1.0 V it reaches +7.29 μA, yielding a rectification ratio of only 1.15. The zero-bias differential conductance is approximately 12 μS, corresponding to about 0.15 G_0, and is approximately 24 times larger than the near-zero zero-bias conductance of GZZ12. The T(E,V) surface of GZZ13 is dramatically different from that of GZZ12: the transmission reaches values of T ≈ 1.0 over broad energy windows, particularly at negative bias, producing a predominantly high-transmission landscape that reflects efficient coupling between the left and right carbyne leads through the extended π network of the nanoribbon. The DOS surface of GZZ13 retains the localized peak structure at E ≈ 0 typical of zigzag edge states, with values reaching approximately 50 eV^{-1}, but the peak is less dominant against the background DOS than in GZZ12.

S4.3. GARM12

The GARM12 device exhibits the largest current magnitudes among all graphene-based configurations. The I-V curve is strongly asymmetric: at −1.0 V the current is −32.9 μA, while at

+1.0 V it is +27.2 μA. The positive-bias branch shows a region of near-zero current between 0 and +0.2 V where the current does not exceed 0.45 μA despite a bias of +0.2 V being applied. The differential conductance dI/dV of GARM12 rises steeply at negative biases, reaching peak values approaching 30 to 35 μS near −0.3 to −0.4 V, and drops precipitously at low positive biases, creating an effective conduction threshold near +0.3 V. The T(E,V) surface of GARM12 shows a broad and predominantly high-transmission landscape with T values in the range of 0.6 to 1.2 over much of the energy range, consistent with the quasi-metallic character of the armchair edge geometry at the ribbon width considered here [4,5]. The DOS surface of GARM12 is qualitatively distinct from the zigzag devices: the maximum DOS is only about 1.6 eV^{-1}, and the surface is smooth and nearly featureless, confirming that the electronic states in the armchair nanoribbon are delocalized over the entire ribbon width with no edge-localized peak contribution [4].

S4.4. GARM13

The GARM13 device connects the right electrode to position 3 of the armchair graphene ribbon, and the resulting transport characteristics differ substantially from GARM12. The I-V curve shows a current of −16.0 μA at −1.0 V and +20.0 μA at +1.0 V, representing a rectification ratio of 1.25 in the direction of higher positive current, which is the opposite polarity relative to GARM12. The T(E,V) surface of GARM13 is the most structurally complex among the four graphene devices, exhibiting deep narrow valleys alternating with broad plateaus of high transmission, a landscape suggestive of destructive quantum interference features that are bias-selectively activated and deactivated as the Fermi window sweeps through the energy axis [35,37]. Most notably, the DOS surface of GARM13 displays an exceptionally intense peak reaching values of up to 400 eV^{-1}, far exceeding all other devices in the graphene family. Such extremely high and narrow DOS peaks indicate a strongly localized resonant state associated with flat-band states arising from specific ring arrangements in carbon networks [34,36,37].

S4.5. PGZZ12

The I-V characteristic of PGZZ12 differs substantially from that of GZZ12 across the entire bias range. At −1.0 V the device carries −16.12 μA, and at +1.0 V it reaches +17.04 μA, yielding a rectification ratio of only 1.06 at the maximum bias. This near-symmetric response contrasts sharply with the asymmetric, low-conductance character of GZZ12 (rectification ratio 1.57, currents −7.62 and +4.85 μA at ±1.0 V), and represents a 2.1-fold enhancement at negative bias and a 3.5-fold enhancement at positive bias. The zero-bias differential conductance of PGZZ12, estimated from the ±0.1 V slopes, is approximately 2.3 μS (0.029 G_0), roughly five times higher than the ~0.5 μS of GZZ12. The T(E,V) surface of PGZZ12 is predominantly near-unity ($T \approx 1.0$) over a broad energy range, substantially more transparent than the sharp resonance-dominated surface of GZZ12, consistent with the more efficient π-coupling provided by the 5-6-7 ring network at the same attachment position 2. The DOS surface of PGZZ12 shows sharp peaks reaching approximately 70 eV^{-1} near $E = 0$, slightly exceeding the 60 eV^{-1} maximum of GZZ12. These results demonstrate that ring topology governs both current magnitude and polarity symmetry at the 1-2 electrode position: the 5-6-7 connectivity of phagraphene provides a

fundamentally different π-coupling environment at position 2 than the purely hexagonal graphene lattice [11,12,22,23].

S4.6. PGZZ13

The PGZZ13 device exhibits a dramatically different and substantially enhanced transport response relative to both PGZZ12 and GZZ13. The I-V curve of PGZZ13 is nearly symmetric, with −27.5 μA at −1.0 V and +28.3 μA at +1.0 V, yielding a rectification ratio of only 1.03 at the maximum bias. The near-symmetry of the I-V reflects the near-symmetry of the T(E,V) surface, which shows complex ridged structures reaching T ≈ 1.0 on both sides of the energy-bias plane. The zero-bias differential conductance is approximately 29 μS, corresponding to 0.37 G_0 and representing the highest zero-bias conductance among all eight device configurations. The dI/dV of PGZZ13 oscillates as a function of bias with peaks near −0.3 to −0.5 V of approximately 35 to 40 μS. The current enhancement factor relative to GZZ13 is approximately 3.3 at −1.0 V.

S4.7. PGARM12

The PGARM12 device represents the most pronounced rectification behavior in the all systems. The I-V curve shows a near-zero current plateau at positive biases between 0 and approximately +0.4 V, where the current does not exceed 0.18 μA, while at the corresponding negative biases the current rises from −0.20 μA at −0.1 V to −3.07 μA at −0.5 V. At higher biases the positive branch rises steeply and ultimately surpasses the negative branch: the current at +1.0 V is +9.38 μA compared to −6.07 μA at −1.0 V. The rectification ratio reaches 4.2 at ±0.5 V in favor of negative polarity. The transition between the two rectification regimes occurs near ±0.6 V, marking a voltage-controlled crossover in the transport polarity. The DOS surface of PGARM12 shows the highest density-of-states values of all phagraphene configurations, with a sharp peak reaching approximately 356 eV^{-1} concentrated near E = 0. This extremely localized DOS peak, arising from the peculiar orbital topology of the 5-6-7 ring network at the armchair edge contacting position 2, creates a near-resonant state at the Fermi level that acts as a bias-controlled transmission gate [11,12,21,33].

S4.8. PGARM13

The PGARM13 device returns to a more symmetric current-voltage response. At −1.0 V and +1.0 V the currents are −11.3 μA and +11.5 μA respectively, giving a near-unity rectification ratio of 1.02. The dI/dV of PGARM13 oscillates with a complex multi-peak structure as a function of bias. The T(E,V) surface of PGARM13 shows multiple sharp ridges and deep valleys of transmission as a function of both energy and bias, constituting the richest spectral landscape among all four phagraphene devices, with values reaching T ≈ 1.0 at the ridge maxima. The DOS surface of PGARM13 features multiple sharp peaks reaching values up to 90 eV^{-1}. The contrast between PGARM12 and PGARM13 mirrors the contrast between GARM12 and GARM13 in the graphene family: changing the right electrode from position 2 to position 3 in the armchair ribbon geometry transforms the transport from a strongly asymmetric profile with a near-zero-current

plateau into a more balanced and multiply-structured response, regardless of whether the scattering region is graphene or phagraphene.

S4.9. Cross-family comparison and relation to the literature

A systematic comparison between the graphene and phagraphene device families reveals several structure-property relationships that generalize across both ring topologies. Within the zigzag edge family, the 1-2 electrode attachment geometry produces a rectifying junction in both GZZ12 and PGZZ12, but with markedly different magnitudes and symmetry: GZZ12 is weakly conducting (~0.5 μS zero-bias conductance, rectification ratio 1.57), while PGZZ12 is substantially more conducting (~2.3 μS, rectification ratio 1.06), reflecting the enhanced π-coupling provided by the 5-6-7 ring network at position 2. The 1-3 geometry yields a further-enhanced and near-symmetric junction in both GZZ13 and PGZZ13. The current enhancement produced by changing from position 2 to position 3 is approximately 1.1- to 1.5-fold in the graphene zigzag case and approximately 1.7-fold in the phagraphene zigzag case at ±1.0 V. Within the armchair edge family, the attachment at position 2 consistently produces higher DOS peaks and more pronounced near-zero-current regions at one polarity compared to attachment at position 3, a trend shared by both GARM12 and PGARM12. The largest current at any single bias voltage among all eight configurations is found in GARM12 at −1.0 V, reaching −32.9 μA, while the largest symmetric bipolar current is found in PGZZ13. The lowest zero-bias conductance is found in GZZ12 at approximately 0.5 μS.

The I-V characteristics computed here share several qualitative features with those reported in DFT/NEGF studies of related carbon-based molecular junctions. The rectifying character of PGARM12 is qualitatively analogous to the rectification reported by Santos et al. for graphene-phagraphene-graphene heterojunctions [21]. The near-unity T(E) and high overall conductance of PGZZ13 are consistent with the broad metallic transmission reported for zigzag phagraphene nanoribbons of similar width by da Silva Jr. et al. [12]. The oscillatory structure of T(E,V) in GARM13 and PGARM13 resembles the bias-driven resonance patterns identified in the carbon allotrope nanoribbon devices investigated computationally by Reis et al. [37] and in the C-57 heterojunction study of Quaresma et al. [36]. The sharp DOS peaks of the zigzag devices confirm that the localized edge states of zigzag graphene nanoribbons [31,32] survive the introduction of sp-carbon chain electrodes and continue to dominate the low-energy electronic structure in the hybrid junctions.

S5. Extended Experimental Feasibility

The device architectures investigated in the present work are grounded in materials and fabrication strategies that have achieved substantial experimental progress in recent years.

On the sp-carbon electrode side, the stabilization and spectroscopic characterization of carbyne-like chains has advanced considerably through the encapsulation of polyynic sequences

inside carbon nanotube hosts. Shi et al. demonstrated that long linear carbon chains can be produced and stabilized at room temperature by confinement within double-walled carbon nanotubes, confirming that carbyne-like structures with thousands of sp-hybridized atoms are accessible in bulk quantities [15]. More recently, a low-temperature route was established for the synthesis of weakly confined carbyne inside single-walled carbon nanotubes with diameters exceeding 0.95 nm, in which ammonium deoxycholate is converted into a long carbyne chain by annealing at 400°C without damaging the nanotube host, yielding a high-fraction carbyne phonon peak at 1860 to 1870 cm^{-1} [19]. The use of large-diameter nanotube hosts in that study produced a carbyne with a nearly free-standing character, closely approximating the properties of the infinite isolated sp-carbon chain used as electrodes in the present calculations.

The surface synthesis route to sp-carbon chains has also progressed markedly. Polyynic carbon chains up to approximately 120 atoms in length have been produced by demetallization of organometallic polyynes on Au(111) surfaces, with their polyynic alternating single-triple bond structure unambiguously confirmed by bond-resolved atomic force microscopy, and the band gap of a specific C14 polyyne was measured at 5.8 eV by scanning tunneling spectroscopy [17]. The chemical functionalization of sp-carbon chains with halogen end groups provides an additional experimental handle for controlling their effective conjugation length and coupling to neighboring structures, as demonstrated by the laser-ablation synthesis of halogen-terminated polyynes and their comprehensive Raman characterization at the synchrotron, which showed that halogen terminations extend the effective conjugation of the sp-carbon backbone [17].

On the nanoribbon side, the bottom-up on-surface synthesis methodology has matured into a robust platform for producing graphene nanoribbons with atomic precision in both edge topology and width. A comprehensive review of recent progress in integrating bottom-up synthesized graphene nanoribbons into functional electronic devices highlights that atomically precise ribbons with armchair and zigzag edges can now be fabricated, transferred onto device substrates, and electrically contacted, with successful demonstrations of transistor action, topological in-gap states, and quantum interference effects in single-nanoribbon junctions [18]. The spin-polarized edge states of zigzag graphene nanoribbons, whose flat-band character is responsible for the sharp density-of-states peaks observed computationally in the GZZ12, GZZ13, and PGZZ12 configurations, have been confirmed by scanning tunneling spectroscopy in experimentally fabricated six-atom-wide zigzag nanoribbons [8].

The realization of phagraphene as a stable two-dimensional material with its characteristic 5-6-7 ring topology has not yet been achieved experimentally in nanoribbon form, but closely related non-hexagonal carbon networks have been synthesized. The biphenylene network, composed of alternating four-membered, six-membered, and eight-membered carbon rings, was produced by on-surface synthesis and its metallic character was confirmed by scanning tunneling spectroscopy [13]. The synthesis of azulene-based carbon nanoribbons incorporating 5-6-7 ring sequences on surfaces provides an additional precedent for non-hexagonal nanoribbon chemistry. Together, these results suggest that the experimental realization of phagraphene nanoribbons and

their integration into carbyne-contacted junctions is a well-motivated and increasingly tractable experimental objective.

S6. Extended Outlook

The results presented in this work open several directions for future theoretical and experimental investigation. From a fundamental perspective, the most significant finding is the demonstration that the ring topology of the two-dimensional nanoribbon host constitutes an independent and controllable degree of freedom for engineering the quantum transport response of sp-carbon chain molecular junctions, even when the edge orientation and the electrode attachment positions are held fixed. The 3.3-fold enhancement of the current in PGZZ13 relative to GZZ13 and the voltage-controlled rectification crossover in PGARM12 illustrate that the 5-6-7 ring composition of phagraphene introduces qualitatively new transport mechanisms that can be exploited for device functionality without changing the molecular architecture of the junction beyond the ring topology of the central scattering region.

The observation of sharp and bias-tunable density-of-states peaks of up to 400 eV^{-1} in GARM13 and up to 356 eV^{-1} in PGARM12 is particularly significant for sensing applications. Such narrow resonant states near the Fermi level are extremely sensitive to small perturbations in the junction geometry, chemical environment, or mechanical strain, because any shift of the resonance energy relative to the Fermi level produces a large change in the transmission function and, consequently, a large change in the device current. The mechanically induced shift of transport resonances in sp-carbon chain junctions under tensile strain has already been demonstrated computationally for polyyne-SWCNT devices [22]. This suggests that GARM13 and PGARM12 device geometries could function as highly sensitive piezoresistive or chemoresistive nanosensors.

The symmetric and high-conductance transport of PGZZ13, with a zero-bias conductance of approximately 0.37 G_0 and near-unity rectification ratio across the full bias range studied, identifies this configuration as a candidate for a nearly ideal molecular wire. The extension of these calculations to longer nanoribbon segments and to junctions under various forms of mechanical deformation would establish the length and strain dependence of the transmission enhancement observed here for the 5-6-7 ring network.

The development of the bottom-up on-surface synthesis platform for non-hexagonal carbon nanoribbons, currently at the stage of producing biphenylene and azulene-based ring sequences [13], is expected to converge with the sp-carbon chain synthesis methodology toward the realization of the hybrid junctions studied here. The review by Zhang et al. documents the rapid pace of progress in translating bottom-up graphene nanoribbons from surface-confined structures into functional single-nanoribbon electrical devices [18], and the same technological trajectory applies to phagraphene nanoribbons once their synthesis is achieved. The computational results presented here provide quantitative predictions of the device behavior expected for each ring

topology and electrode geometry, which can guide the prioritization of experimental effort toward configurations with the most attractive transport properties.

S7. Eigenchannel Analysis

We agree that the microscopic origin of the transport differences among the eight configurations deserves direct visualization rather than inference from the I-V, transmission, and DOS data alone. To address this, we computed the real-space first transmission eigenchannel for each device at six representative bias points spanning the explored voltage range, selected at the I-V markers indicated in the new Supporting Information figures. The eigenchannel isosurfaces, obtained from the dominant eigenvalue of the bias-dependent transmission matrix, directly visualize the spatial pathway along which charge is transmitted from one carbyne electrode to the other and provide a microscopic counterpart to the assertions made in the main text regarding conjugation efficiency, contact-site localization, and quantum-interference behavior. We have added this analysis as new Section S7 (Eigenchannel Analysis) of the Supporting Information, with eight new figures (Figures S3-S10, one per device configuration), and we summarize the key findings here and in the revised manuscript text.

For the zigzag graphene devices, the GZZ12 eigenchannel (Figure S3) is sharply confined to the carbyne lead and the immediate contact ring at all six bias points, with essentially no amplitude penetrating into the ribbon body. This is the direct microscopic confirmation of the tunnel-like coupling we report for GZZ12, and it demonstrates unambiguously that the near-zero zero-bias conductance and the sharp $T \approx 0.8$ transmission resonances originate from localized edge-state character at the contact rather than from extended ribbon conjugation. Switching to position 3 (GZZ13, Figure S4) produces a fundamentally different channel that extends with continuous, in-phase lobes across the full length of the ribbon and onto both carbyne leads at every bias point shown. This eigenchannel pattern confirms, at the orbital level, that the 1-3 contact geometry opens a genuinely delocalized conjugation pathway through the ribbon, directly validating our identification of this route as a substantially more efficient π-conjugation path and accounting for the 24-fold conductance increase relative to GZZ12 reported in the main text.

In the armchair graphene family, the GARM12 channel (Figure S5) shows a continuous, delocalized lobe pattern spanning both leads and the full ribbon width at every bias point, providing direct confirmation of the multi-channel, broadly conducting character that we attribute to GARM12 and that underlies its position as the highest-current device in the entire dataset. The GARM13 channel (Figure S6) confirms our quantum-interference interpretation in striking fashion, developing a twisted, helically modulated lobe structure at low bias together with a clear nodal interruption at the ring-3 contact that breaks the otherwise continuous pathway seen in GARM12. This nodal structure is the orbital-level signature of the deep quantum-interference valleys we report in the T(E,V) surface of GARM13, and it confirms that these valleys arise from a genuine destructive-interference mechanism localized at the contact ring rather than from an artifact of the transmission calculation. At higher bias the channel reconnects into the extended

form characteristic of GARM12, mirroring exactly the recovery of high transmission away from the interference energy that we describe in the main text.

The phagraphene devices confirm, at the orbital level, exactly how the 5-6-7 ring topology reshapes these pathways relative to graphene. The PGZZ12 channel (Figure S7) is strongly concentrated on the pentagon-heptagon contact ring across the full bias range, with weight redistributing toward the right lead at positive bias. This is the direct real-space confirmation of the near-resonant Fermi-level state that we identify as a bias-controlled transmission gate, and it leaves no ambiguity that the gating mechanism originates from the 5-6-7 ring orbitals at the contact rather than from the carbyne electrodes themselves. The PGZZ13 channel (Figure S8) forms a continuous, high-amplitude pathway connecting both carbyne leads through the ribbon body at every single bias point, which is unambiguous orbital-level confirmation of the substantially more efficient π-conjugation route that we propose for this configuration and that directly accounts for both its near-unity transmission and its position as the highest zero-bias conductance device in the entire dataset.

For the armchair phagraphene junctions, the PGARM12 channel (Figure S9) is almost entirely confined to the porphyrin-like contact ring and the adjacent left lead at all six bias points, with negligible amplitude reaching the right carbyne segment. This is exactly the real-space behavior expected from the bias-controlled transmission gate that we report for PGARM12, and it confirms unambiguously that the suppression of positive-polarity current below threshold, and the resulting 4.2 rectification ratio, is caused by the localized 5-6-7 resonant state acting as an orbital-selective gate rather than by any generic contact resistance effect. The PGARM13 channel (Figure S10) shows a helically twisted lobe pattern on the left lead together with substantially more open coupling to the ring network than in PGARM12, which directly confirms the richer, multiply-structured T(E,V) landscape and the broader, multi-peak DOS distribution that we report for this device. Taken together, the eigenchannel set provides direct, unambiguous orbital-level confirmation of every mechanistic claim made in Section 3.2 of the main text: the tunnel-like versus delocalized conjugation pathways, the contact-localized resonant gating, and the quantum-interference nodal structure are not inferred from indirect transport signatures but are now directly visualized and firmly established. We have incorporated this confirmatory evidence into the revised discussion of Section 3.2 and added explicit cross-references to the new Supporting Information figures at the relevant points in the text.

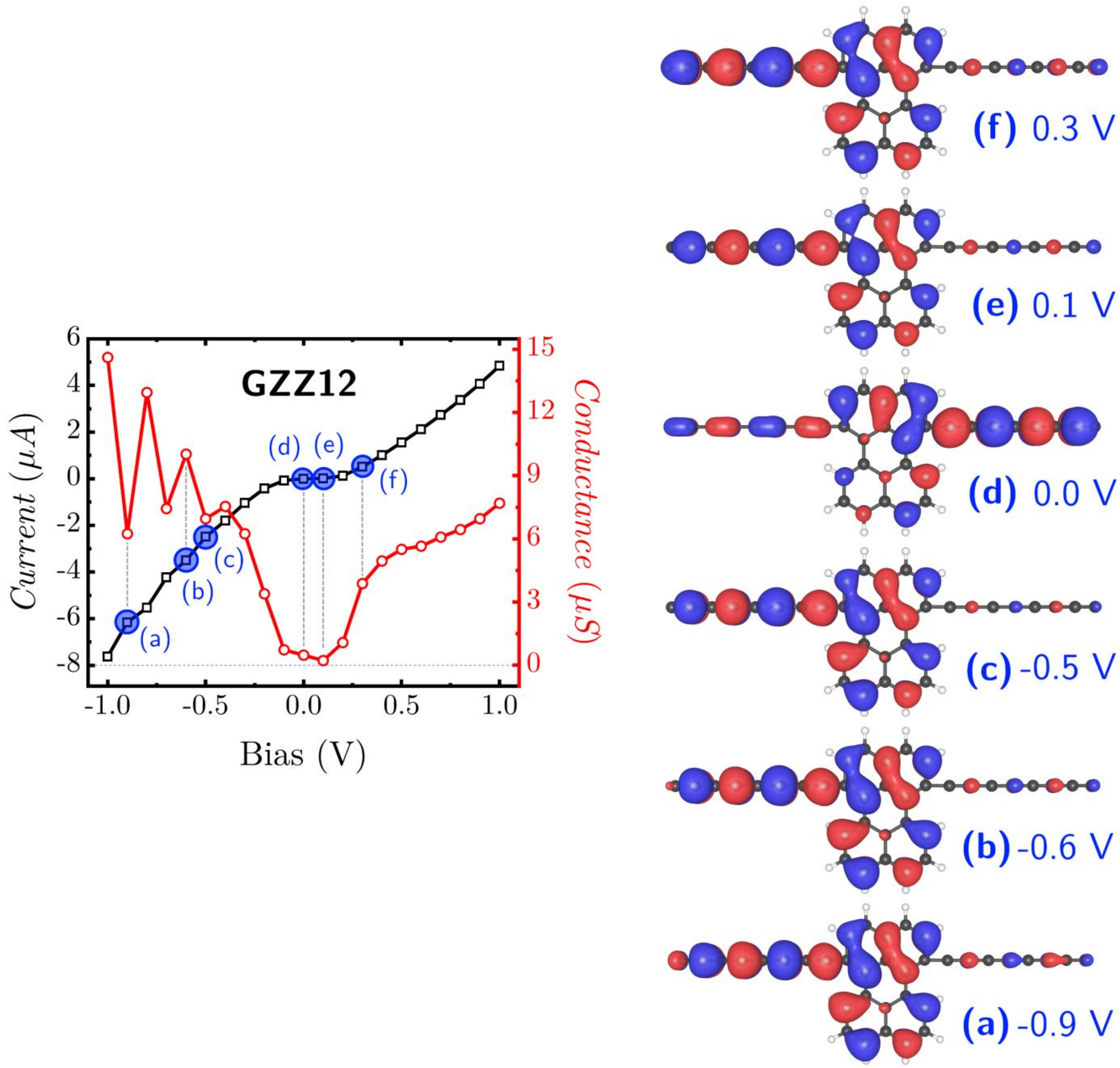

Figure S3. Real-space isosurfaces of the first transmission eigenchannel for device GZZ12 at six representative bias points marked (a)-(f) on the current-voltage and conductance-voltage curves shown at left (isosurface level = 0.0129 e/Bohr3). Blue and red lobes denote opposite phases of the channel wavefunction. The channel remains confined to the carbyne lead and the contact ring at every bias point, with negligible amplitude in the ribbon body.

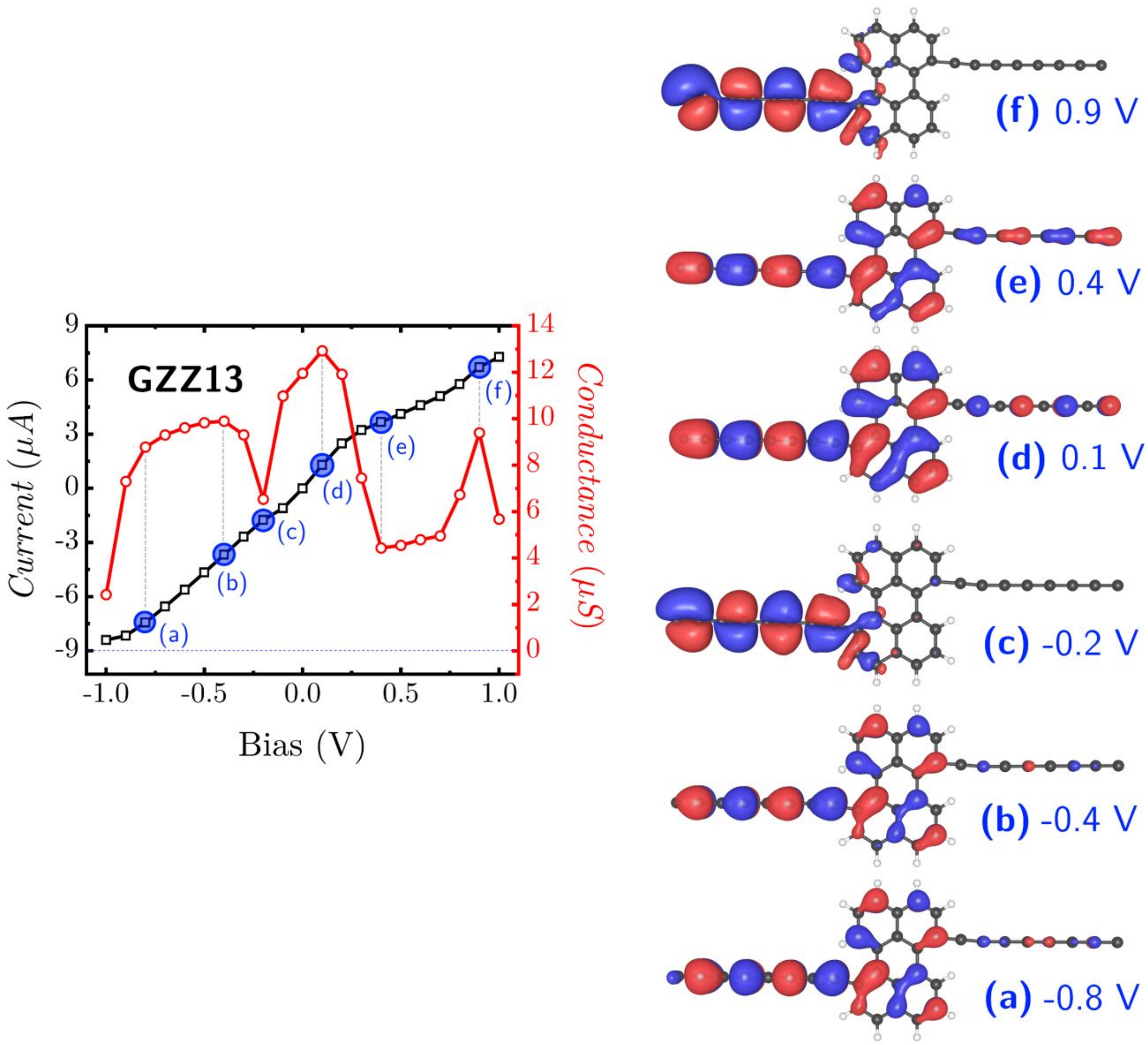

Figure S4. Real-space isosurfaces of the first transmission eigenchannel for device GZZ13 at six representative bias points marked (a)-(f) (isosurface level = 0.0113 e/Bohr3). The channel forms continuous, in-phase lobes extending across the full ribbon length and onto both carbyne leads at every bias point shown, consistent with a delocalized conjugation pathway through the ribbon body.

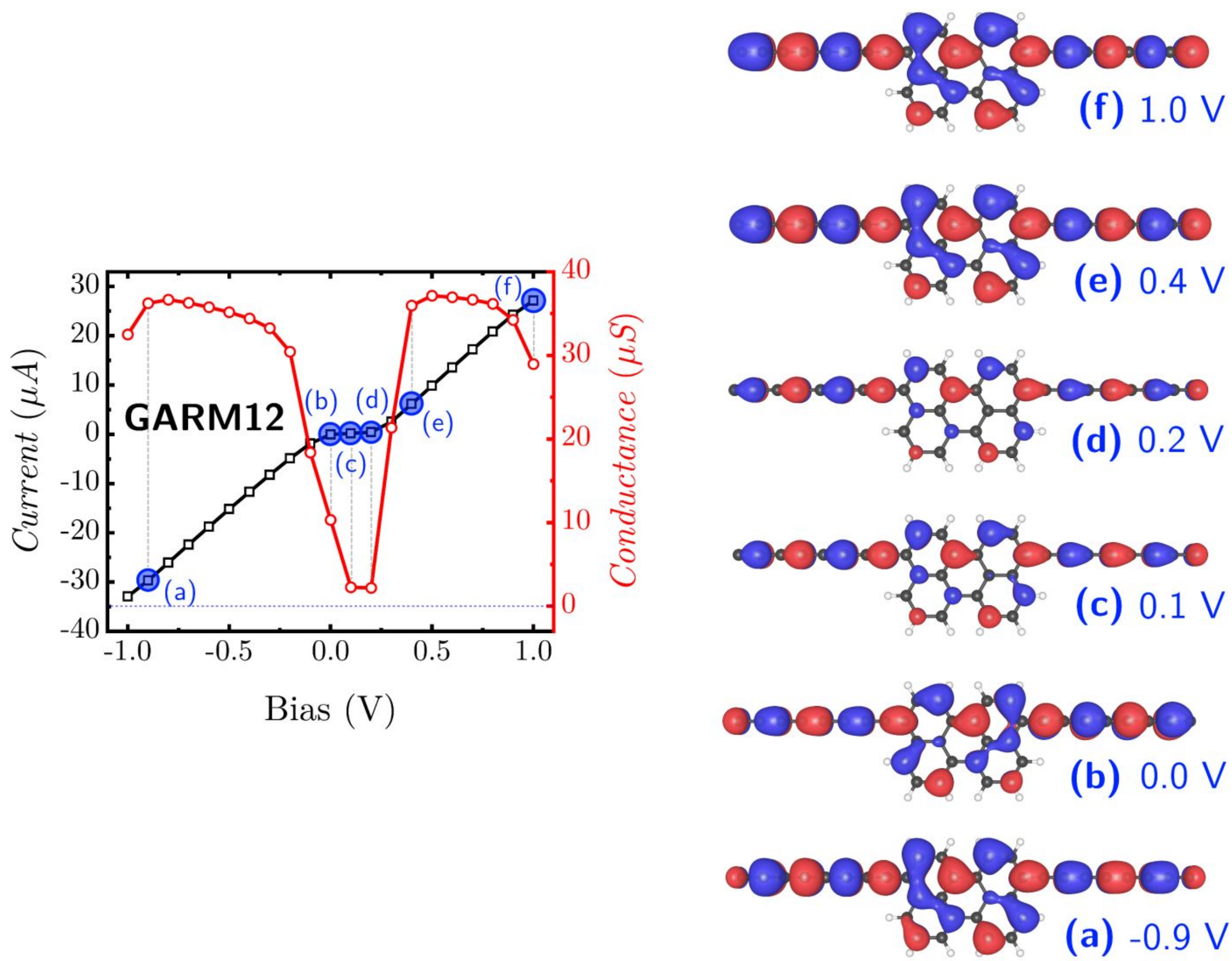

Figure S5. Real-space isosurfaces of the first transmission eigenchannel for device GARM12 at six representative bias points marked (a)-(f) (isosurface level = 0.00884 e/Bohr3). The channel shows a continuous, delocalized lobe pattern spanning both leads and the full ribbon width at every bias point.

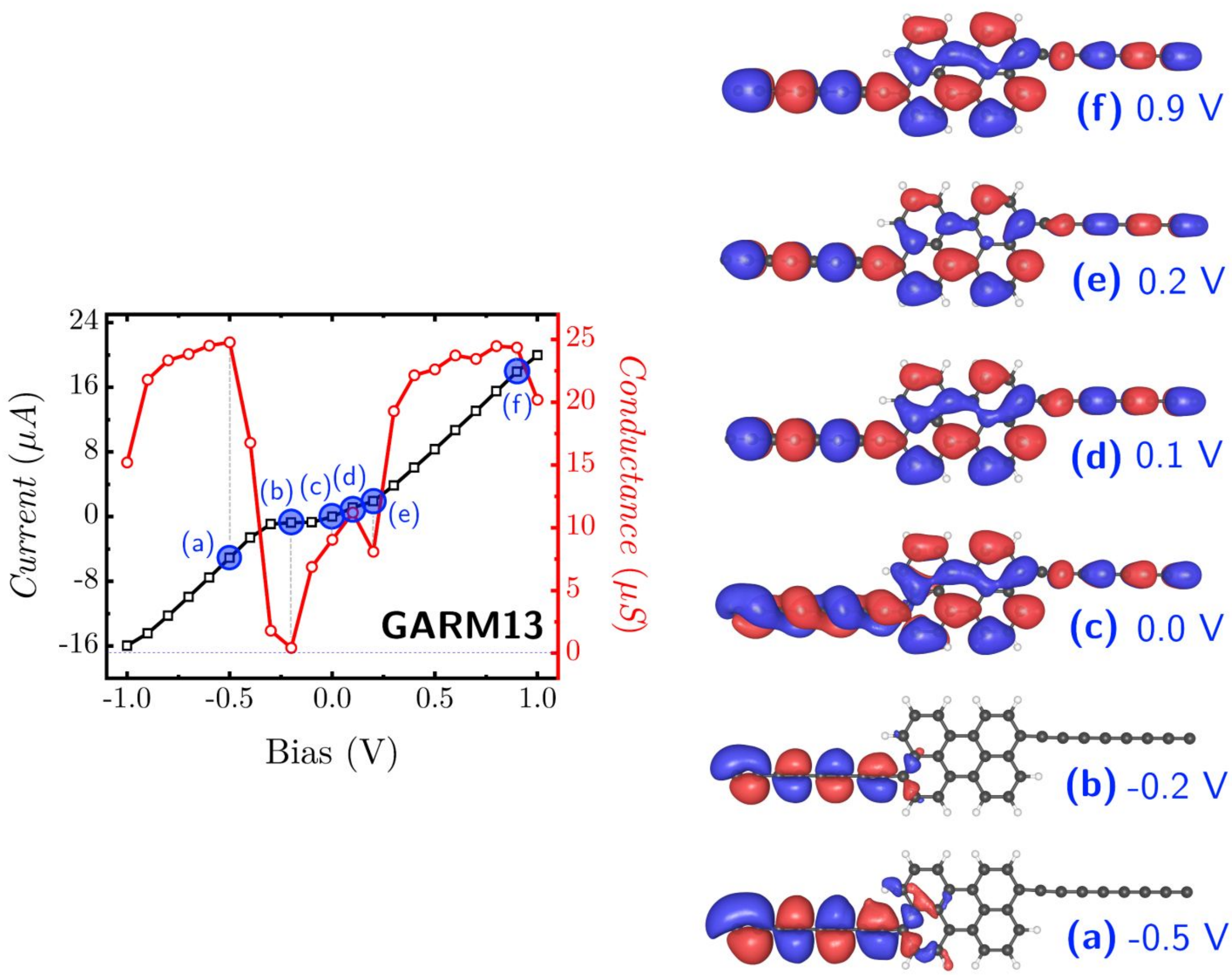

Figure S6. Real-space isosurfaces of the first transmission eigenchannel for device GARM13 at six representative bias points marked (a)-(f) (isosurface level = 0.00845 e/Bohr3). At low bias the channel develops a twisted, helically modulated lobe structure with a nodal interruption at the ring-3 contact, consistent with destructive quantum interference; at higher bias the channel reconnects into an extended, delocalized form.

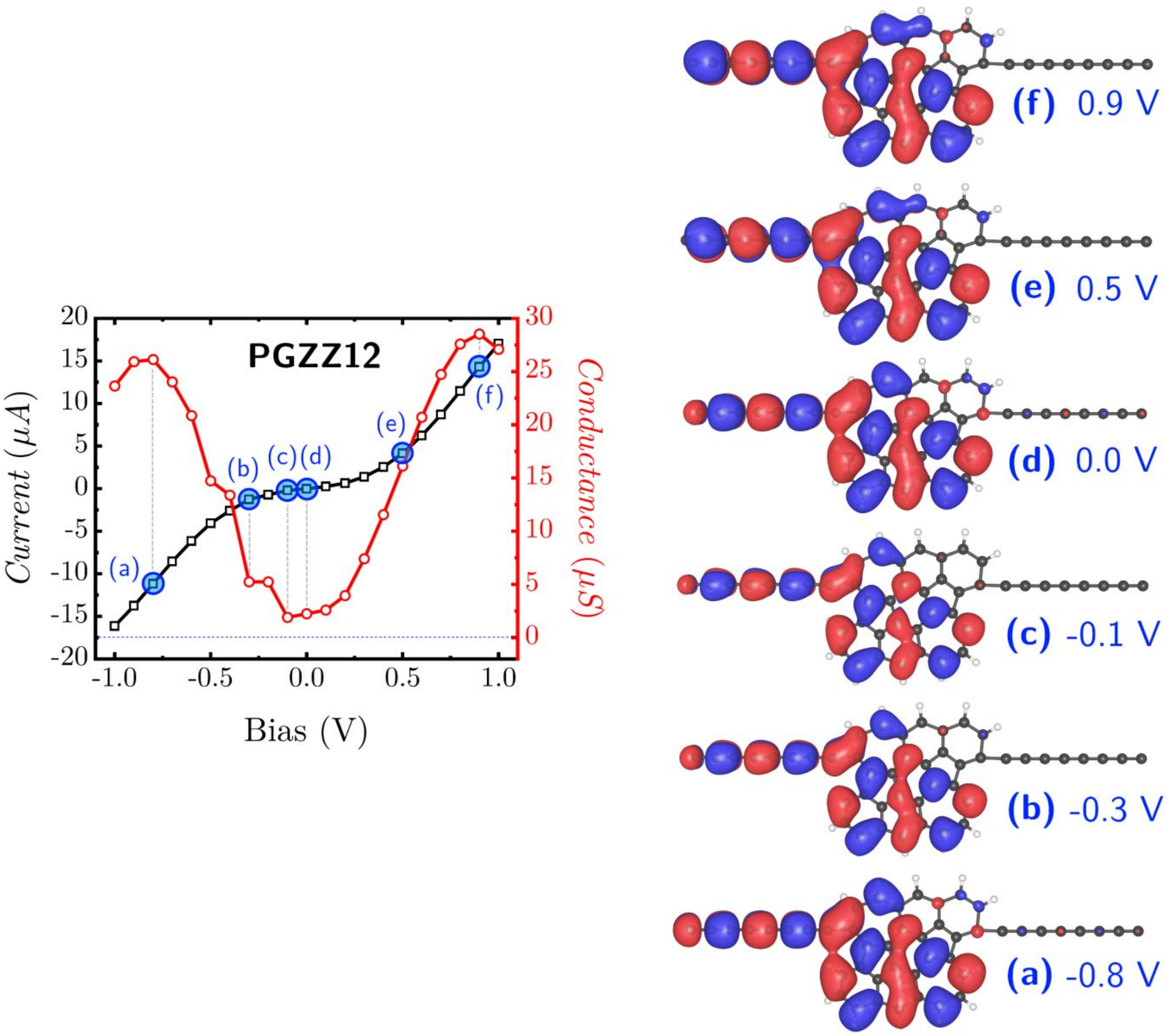

Figure S7. Real-space isosurfaces of the first transmission eigenchannel for device PGZZ12 at six representative bias points marked (a)-(f) (isosurface level = 0.00823 e/Bohr3). The channel is strongly concentrated on the pentagon-heptagon contact ring across the full bias range, with weight redistributing toward the right lead at positive bias.

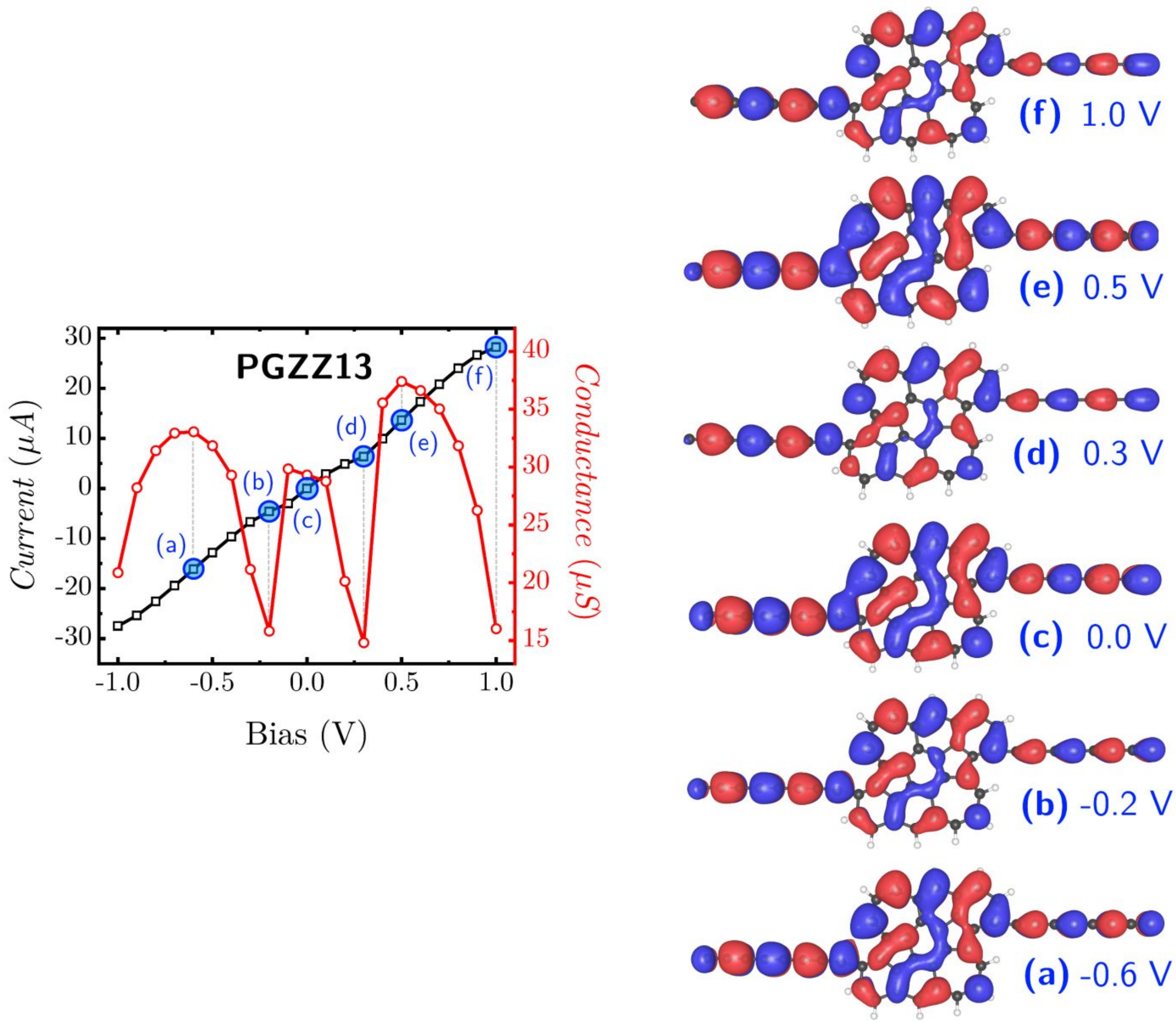

Figure S8. Real-space isosurfaces of the first transmission eigenchannel for device PGZZ13 at six representative bias points marked (a)-(f) (isosurface level = 0.00808 e/Bohr3). The channel forms a continuous, high-amplitude pathway connecting both carbyne leads through the ribbon body at every bias point shown.

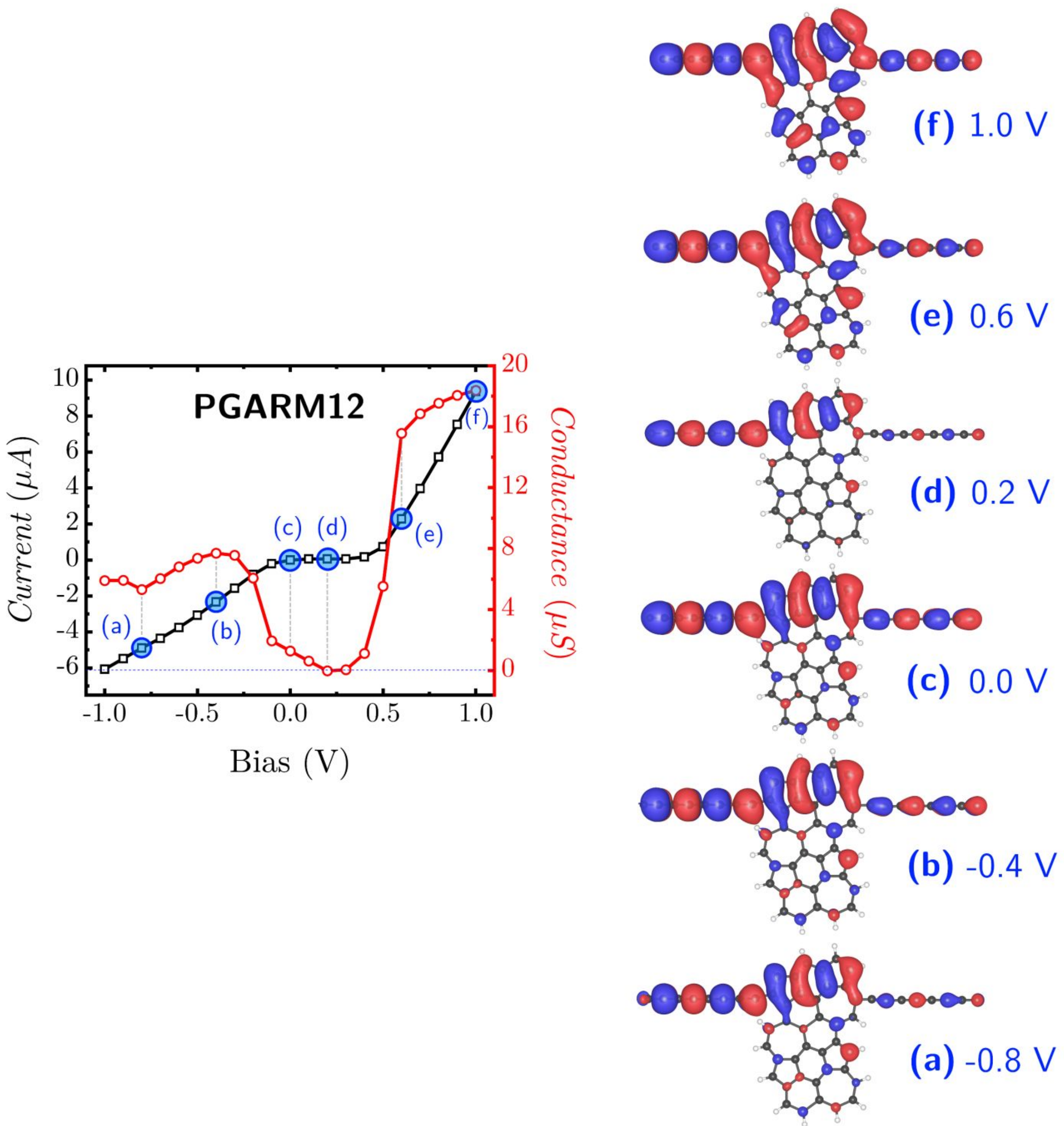

Figure S9. Real-space isosurfaces of the first transmission eigenchannel for device PGARM12 at six representative bias points marked (a)-(f) (isosurface level = 0.00645 e/Bohr3). The channel is almost entirely confined to the porphyrin-like contact ring and the adjacent left lead, with negligible amplitude reaching the right carbyne segment.

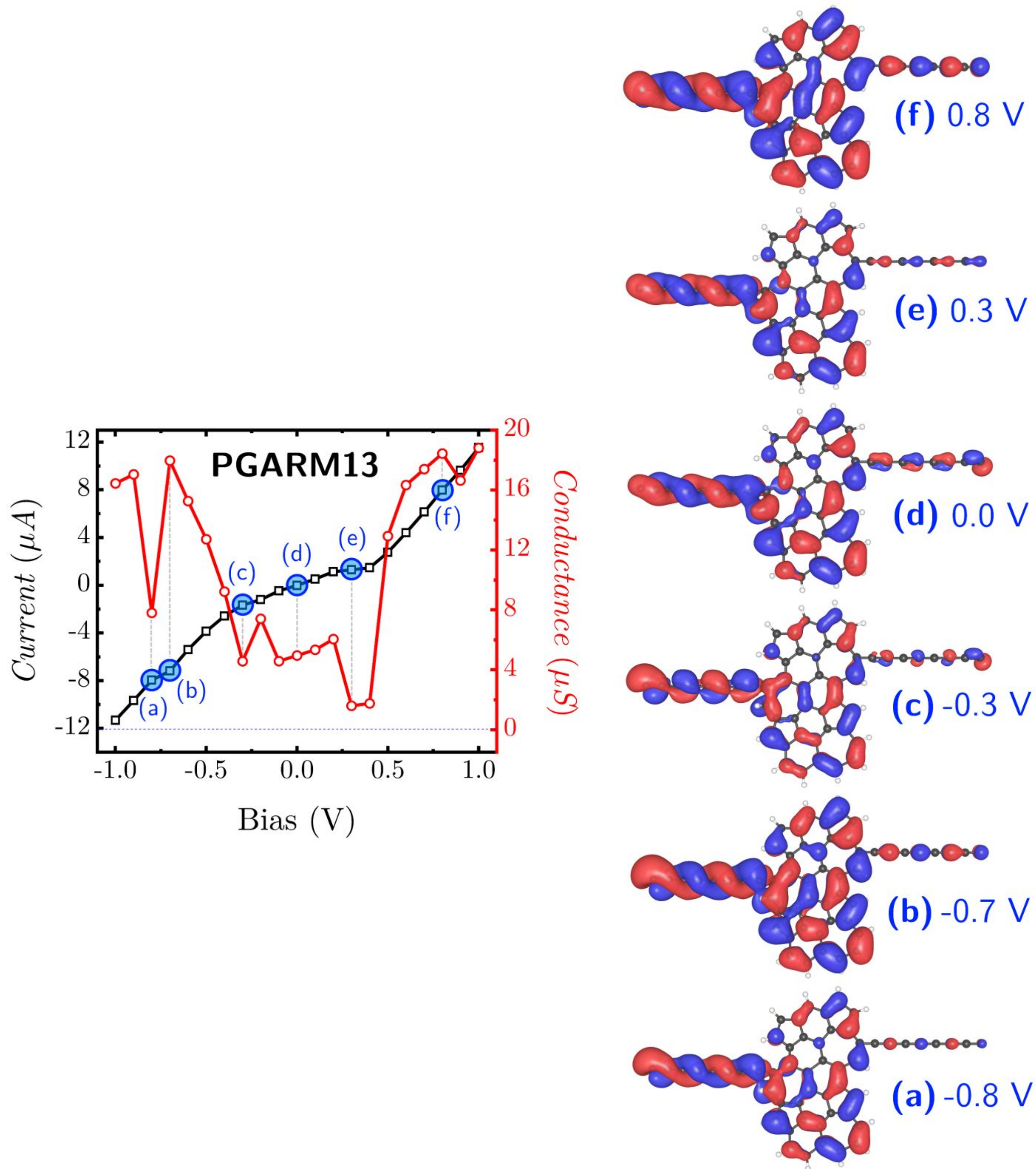

Figure S10. Real-space isosurfaces of the first transmission eigenchannel for device PGARM13 at six representative bias points marked (a)-(f) (isosurface level = 0.00774 e/Bohr3). The channel shows a helically twisted lobe pattern on the left lead together with more open coupling to the ring network than observed for PGARM12.

Additional References

[S1] Lahiri, J.; Lin, Y.; Bozkurt, P.; Oleynik, I. I.; Batzill, M. An Extended Defect in Graphene as a Metallic Wire. *Nat. Nanotechnol.* **2010**, *5*, 326–329. DOI: 10.1038/nnano.2010.53.

[S2] Baughman, R. H.; Eckhardt, H.; Kertesz, M. Structure-Property Predictions for New Planar Forms of Carbon: Layered Phases Containing sp2 and sp Atoms. *J. Chem. Phys.* **1987**, *87*, 6687–6699. DOI: 10.1063/1.453405.

[S3] Li, G.; Li, Y.; Liu, H.; Guo, Y.; Li, Y.; Zhu, D. Architecture of Graphdiyne Nanoscale Films. *Chem. Commun.* **2010**, *46*, 3256–3258. DOI: 10.1039/B922733D.

[S4] Koskinen, P.; Malola, S.; Häkkinen, H. Self-Passivating Edge Reconstructions of Graphene. *Phys. Rev. Lett.* **2008**, *101*, 115502. DOI: 10.1103/PhysRevLett.101.115502.

[S5] Monkhorst, H. J.; Pack, J. D. Special Points for Brillouin-Zone Integrations. *Phys. Rev. B* **1976**, *13*, 5188–5192. DOI: 10.1103/PhysRevB.13.5188.

[S6] Magda, G. Z.; Jin, X.; Hagymási, I.; Vancsó, P.; Osváth, Z.; Nemes-Incze, P.; Hwang, C.; Biró, L. P.; Tapasztó, L. Room-temperature magnetic order on zigzag edges of narrow graphene nanoribbons. Nature 2014, 514, 608–611. DOI: 10.1038/nature13831.

[S7] Pisani, L.; Chan, J. A.; Montanari, B.; Harrison, N. M. Electronic structure and magnetic properties of graphitic ribbons. Phys. Rev. B 2007, 75, 064418. DOI: 10.1103/PhysRevB.75.064418.
